# Polycomb group proteins in cancer: multifaceted functions and strategies for modulation

**DOI:** 10.1093/narcan/zcab039

**Published:** 2021-10-04

**Authors:** Sijie Wang, Sandra C. Ordonez-Rubiano, Alisha Dhiman, Guanming Jiao, Brayden P Strohmier, Casey J Krusemark, Emily C Dykhuizen

**Affiliations:** Department of Medicinal Chemistry and Molecular Pharmacology, College of Pharmacy, Purdue University and Purdue University Center for Cancer Research, 201 S. University St., West Lafayette, IN 47907 USA; Department of Medicinal Chemistry and Molecular Pharmacology, College of Pharmacy, Purdue University and Purdue University Center for Cancer Research, 201 S. University St., West Lafayette, IN 47907 USA; Department of Medicinal Chemistry and Molecular Pharmacology, College of Pharmacy, Purdue University and Purdue University Center for Cancer Research, 201 S. University St., West Lafayette, IN 47907 USA; Department of Medicinal Chemistry and Molecular Pharmacology, College of Pharmacy, Purdue University and Purdue University Center for Cancer Research, 201 S. University St., West Lafayette, IN 47907 USA; Department of Medicinal Chemistry and Molecular Pharmacology, College of Pharmacy, Purdue University and Purdue University Center for Cancer Research, 201 S. University St., West Lafayette, IN 47907 USA; Department of Medicinal Chemistry and Molecular Pharmacology, College of Pharmacy, Purdue University and Purdue University Center for Cancer Research, 201 S. University St., West Lafayette, IN 47907 USA; Department of Medicinal Chemistry and Molecular Pharmacology, College of Pharmacy, Purdue University and Purdue University Center for Cancer Research, 201 S. University St., West Lafayette, IN 47907 USA

## Abstract

Polycomb repressive complexes (PRCs) are a heterogenous collection of dozens, if not hundreds, of protein complexes composed of various combinations of subunits. PRCs are transcriptional repressors important for cell-type specificity during development, and as such, are commonly mis-regulated in cancer. PRCs are broadly characterized as PRC1 with histone ubiquitin ligase activity, or PRC2 with histone methyltransferase activity; however, the mechanism by which individual PRCs, particularly the highly diverse set of PRC1s, alter gene expression has not always been clear. Here we review the current understanding of how PRCs act, both individually and together, to establish and maintain gene repression, the biochemical contribution of individual PRC subunits, the mis-regulation of PRC function in different cancers, and the current strategies for modulating PRC activity. Increased mechanistic understanding of PRC function, as well as cancer-specific roles for individual PRC subunits, will uncover better targets and strategies for cancer therapies.

## INTRODUCTION TO POLYCOMB GROUP PROTEINS

**Figure 1.**
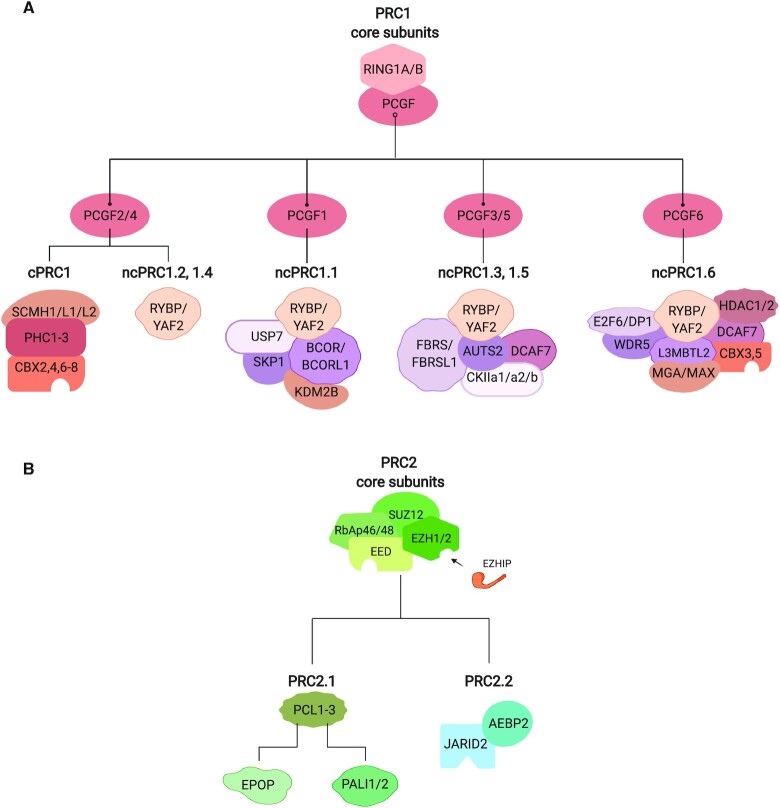
The subunit composition of Polycomb repressive complexes. (**A**) Subunit composition of canonical PRC1 (cPRC1) and noncanonical PRC1 (ncPRC1). (**B**) Subunit composition of PRC2.

The proper regulation of chromatin structure during development is important for establishing and maintaining cell-type specific transcriptional programs. Compared to unicellular organisms, metazoans have an increased number of chromatin regulators, which play essential roles in cell-type specification. One such set of chromatin regulators are the Polycomb group (PcG) proteins, which were originally discovered in *Drosophila* and named for their roles in leg development (1). Subsequently, PcG proteins were identified as crucial regulators of development in vertebrates, acting primarily to modify histones and reduce DNA accessibility (2,3). PcG proteins fall into two broad complexes: Polycomb Repressive Complex 1 and 2 (PRC1 and PRC2) (Figure 1) (4). PRC1 is defined by a RING subunit, which is capable of H2AK119 monoubiquitination, while PRC2 is defined by an EZH subunit, which is capable of H3K27 methylation. Both complexes incorporate a variety of mutually exclusive subunits giving rise to an array of diverse complexes with distinct biochemical activities (5,6).

### Polycomb complex composition

All PRC1s contain a RING ubiquitin ligase subunit with one of six possible PCGF paralogs (Figure 1A). PRC1s that contain CBX and PHC subunits are defined as ‘canonical’ PRC1 (cPRC1), and exclusively incorporate PCGF2 or PCGF4, while PRC1s that contain the RYBP/YAF2 subunit are defined as ‘non-canonical’ PRC1 (ncPRC1) and can contain any PCGF paralog (Figure 1A) (7). The incorporation of different PCGF variants defines ncPRC subcomplexes with different accessory subunits (7,8).

All PRC2s contain an EZH methyltransferase subunit, EED, SUZ12 and RbAp46/48 (Figure 1B) and catalyze mono/di/trimethylation of lysine 27 of histone H3 (H3K27me, H3K27me2, H3K27me3) (9,10). PRC2 can be categorized as PRC2.1 and PRC2.2 depending on the accessory subunits PCL1-3 (PRC2.1) or AEBP2 and JARID2 (PRC2.2), which are involved in genome targeting (11–14).

### Establishment of Polycomb sites

**Figure 2.**
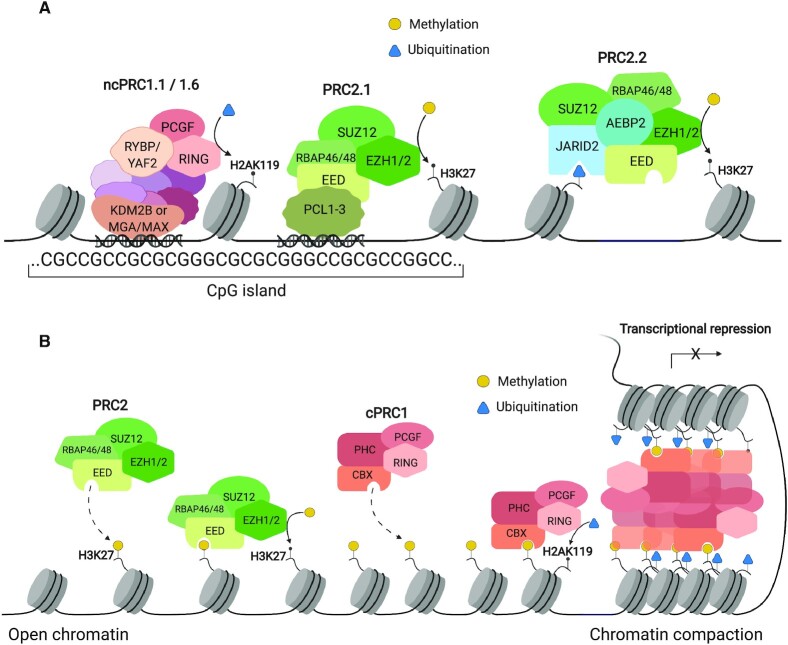
Transcriptional repression by PRCs. (**A**) Initiation. ncPRC1 targets specific DNA sequences (hypomethylated CpG islands) and ubiquitinates H2AK119. H2AK119ub1 is recognized by JARID2 and AEBP2 of PRC2.2, leading to the recruitment of PRC2 and deposition of trimethylation on H3K27. PRC2.1 also associates with hypomethylated CpG islands via PCL-mediated DNA binding and deposits additional H3K27me3. This leads to an initial H3K27me3-mediated compaction, drawing nucleosomes closer for EED to perform allosteric activation of EZH activity. (**B**) Propagation/Maintenance. H3K27me3 binds to aromatic cage of EED, and allosterically activates EZH methyltransferase activity, leading to further trimethylation of nearby unmodified H3K27. H3K27me3 is recognized by the CBX subunit within cPRC1. cPRC1 recruitment leads to further chromatin compaction and transcriptional repression.

Mammals lack the so-called Polycomb Response Elements (PREs) found in lower organisms (15,16); instead, ncPRC1 localizes to unmethylated CpG islands via accessory proteins and catalyzes monoubiquitination of H2AK119 (Figure 2A) (17–20). PRC2.2 subunits JARID2/AEBP2 bind a subset of H2AK119ub1 deposited primarily by ncPRC1 (21,22), while PRC2.1 binds unmethylated CpG islands, at least in part, through PCL association with DNA (23,24). The two PRC2 variants cooperate in establishing sites of H3K27me3 across the genome (25), which can repress transcription in part by blocking acetylation of H3K27. Whether H2AK119ub1 can be transcriptionally repressive at sites without PRC2 activity is not known, as ncPRC1 subunits have also been implicated in gene activation (26–33).

### Polycomb propagation and chromatin compaction

What is sometimes referred to as ‘canonical’ Polycomb function is a mechanism by which Polycomb marks are propagated and maintained (Figure 2B). This is initiated by the association of the PRC2 subunit EED with H3K27me3, which allosterically activates methyltransferase activity of EZH towards unmodified H3K27 at neighboring and non-neighboring nucleosomes (34,35). cPRC1 binds a subset of H3K27me3 via the N-terminal chromodomain of the CBX subunit, monoubiquitinates H2AK119 through the E3 ligase domain of RING1A/B, and oligomerizes via the PHC and CBX subunits to physically compact chromatin (36) into large domains called Polycomb bodies (37). Formation of Polycomb bodies is dependent on PRC2 (38) and cPRC1, in particular the chromodomain of the CBX subunit (39,40) and the sterile alpha motif (SAM) oligomerization domain of PHC (41). These nuclear foci are spatially separated from regions of active transcription as well as regions of constitutive heterochromatin (42). Within these large 3D domains, PcG-bound regions make contacts with other PcG-bound regions several megabases away, resulting in repression across the domain (33). In mammalian cells, Polycomb body distribution changes during differentiation (40,43), as well as transformation (42).

### Maintenance of Polycomb sites

After Polycomb binding sites are established, PcG proteins maintain these sites during cell division. The loss of ncPRC1 (RYBP deletion) in mouse embryonic stem cells (mESCs) can be compensated for with elevated EED, H3K27me3, and CBX7 at shared target loci, indicating some redundancy for ncPRC1 by PRC2/cPRC1 in the maintenance of previously established Polycomb binding sites (21). While PRC2/cPRC1 is not required for initiating Polycomb binding sites, it is critical for transcriptional repression (17) and may be sufficient for Polycomb maintenance/memory, at least in some cell types (44). During differentiation, however, dynamic changes in PRC subunit composition and genome localization require establishment of new Polycomb sites (45).

### Biochemical functions of individual PRC1 and PRC2 subunits

Advanced proteomic techniques have begun to identify the combinations of individual PRC subunits unique to particular cell types. While structural and functional studies have elucidated the mechanistic contribution of critical core subunits, the biochemical contribution of accessory and substoichiometric subunits, and the unique biochemical contribution of individual paralogs are not always clear. A summary of defined subunit domains and known biochemical functions is provided in Table 1.

**Table 1.** Biochemical functions of PRC subunits

Subunit	Constituent motifs and domains (InterPro and CanSAR database)	PDB structures (including partial structures)	Major biochemical function	Additional structural, allosteric or regulatory functions if any known
**PRC1**				
RING1A RING1B (RING2/RNF2)	N-terminal RING-type zinc finger C-terminal RAWUL domain	RING1B	RING1B is the major E3 ubiquitin ligase mediating H2AK119ub1, since RING1B loss has a much larger effect on ubiquitination loss than RING1A (21,46) RING1A stimulates the E3 ligase activity of RING1B containing complexes as seen by *in vitro* reconstitution and ubiquitination assays (47)	RING domain-mediated heterodimerization with PCGF enhances interaction with E2 ubiquitin-conjugating enzyme (48–50) RAWUL domain-mediated interaction with either CBX or RYBP/YAF2 results in different PRC1 assembly (51) RING can dimerize with different PCGF paralogs making a scaffold for distinct PRC1 assembly (52)
PCGF1 PCGF2 (MEL18) PCGF3 PCGF4 (BMI1) PCGF5 PCGF6	N-terminal RING-type zinc finger C-terminal RAWUL domain (PCGF6 lacks the RAWUL domain)	PCGF1 PCGF4 PCGF5 PCGF6	Forms RING domain-mediated heterodimer with RING1 protein and enhances E3-ligase activity (48,49) PCGF2/4 are present in canonical PRC1 while non-canonical PRC1 can contain PCGF1-6 (7)	Structural studies have shown differences in the binding partners of RING1B RAWUL domain and PCGF RAWUL domain (53)
CBX2 CBX4 CBX6 CBX7 CBX8	N-terminal Chromodomain AT hook (CBX2) or AT-like hook (CBX4,6,7,8) C-terminal Pc Box (also called CBX family C-terminal motif)	CBX2 CBX4 CBX6 CBX7 CBX8	Chromodomain-mediated binding to H3K27me3 (or in some cases H3K9me3), positioning the trimethyl mark within an aromatic cage (54) Pc box-mediated interaction with RING1 (55) Chromodomain-mediated non-specific binding to nucleic acids in CBX4, 6–8 (56) CBX8 chromodomain simultaneously associates with both DNA and H3K27me3 (57) CBX2 participates in nucleosome binding and phase separation through a serine rich patch (58) CBX4 has an E3 SUMO ligase activity which has been shown to enhance sumoylation of CtBP, DNMT3a, BMI-1 (59–61)	CBX paralogs have high sequence similarity in the chromodomain and Pc box, yet displaying low similarity outside these domains indicating roles in paralog-specific functions (54,58,62,63) CBX2, 6 and 8 compact nucleosomal arrays in the absence of PRC1 complex, but CBX7 fails to show such compaction. This compaction (and hence, transcriptional repression) is promoted by a highly basic region absent in CBX7 (36) CBX2 undergoes phosphorylation at Ser42 within the chromodomain which alters its binding specificity from H3K27me3 to H3K9me3 *in vitro* (64) CBX8 has been implicated in transcriptional activation through interactions with activators like AF9/ENL implying functions outside cPRC1 (62,65–67)
PHC1 PHC2 PHC3	FCS-type zinc finger C-terminal Sterile alpha motif (SAM)	PHC1 PHC3	SAM-mediated oligomerization with self or other SAM containing proteins, facilitating large scale compaction by phase separation (42,68,69) Such condensates also display enhanced H2A ubiquitination (68)	
SCMH1 SCML1 SCML2 SCML4	MBT repeats SLED domain SAM	SCMH1 SCML2	Oligomerization via SAM with other SAM containing proteins (70,71)	Associates with PRC1 at sub-stoichiometric levels (72,73)
RYBP YAF2	N-terminal zinc finger Yaf2/RYBP C-terminal binding motif	RYBPYAF2	RYBP positively modulates the E3 ligase activity of RING1B by stabilizing RING1B protein levels in some cell types (74). However, in mESCs, depletion of RYBP was observed not to affect RING1B protein level (75) RYPB and YAF2 enhance RING1B-PCGF1 mediated H2A ubiquitination activity *in vitro*. (75) RYBP/YAF2 binds H2AK119ub1 and promotes propagation and deletion of RYBP/YAF2 reduces H2AK119ub1 levels at Polycomb target sites (76)	RING1-RYBP/YAF2 interaction is mutually exclusive with RING1-CBX (7) RYBP/YAF2 containing ncPRC1 exhibits stronger *in vitro* nucleosome monoubiquitination activity than CBX-containing cPRC1 (77)
KDM2B	N-terminal Jumonji (JmjC) domain CXXC-type zinc finger PHD-type zinc finger C-terminal F-box domain	KDM2B	Major subunit for targeting to unmethylated CpG islands (77) JmjC-mediated H3K4 and H3K36 demethylase activity for transcriptional repression (78,79)	
BCOR BCORL1	Non-ankyrin repeat domain Ankyrin repeats BCOR(L)-PCGF1 binding domain	BCOR BCORL1	N-terminal mediated interaction with other proteins like CtBP1, HSPD1 and BCL6 (80) C-terminal mediated binding to PCGF1 to form the binding surface for KDM2B incorporation (81)	
MAX MGA	bHLH DNA binding domain	MAX	bHLH-mediated binding to E-box sequences recognized by Myc, acting as DNA targeting subunits (82) MAX and MGA form a heterodimer for binding to E-box sites (83)	MGA additionally contains a conserved DNA binding T-box or T-domain (83)
E2F6 DP1 DP2	N-terminal E2F/DP family winged helix DNA binding domain C-terminal E2F Transcription factor CC-MB domain (E2F6) C-terminal Transcription factor DP domain (DP1, DP2)	DP1 DP2	E2F6 interacts with DP1 or DP2 to bind to E2F recognition sequences, acting as DNA targeting subunits (84)	
L3MBTL2	N-terminal FCS-type zinc finger MBT repeats	L3MBTL2	MBT repeats bind H3 and H4 mono- and di-methylated tails *in vitro* (85)	The methyl binding function has been shown to be dispensable for repression (85)
AUTS2			Shown to be transcriptionally activating at target loci (28)	AUTS2 containing ncPRC1.5 has been shown to have reduced H2AK119 ubiquitination activity *in vitro* and AUTS2-RING1B co-bound genomic targets exhibit lower levels of H2AK119ub1 and H3K27me3 in mouse brain cells (28)
**PRC2**				
EZH1 EZH2	N-terminal WD repeat binding domain SANT domain CXC domain C-terminal SET domain	EZH1 EZH2	Catalytic subunit with SET domain-mediated H3K27 methylation activity using S-Adenosyl Methionine (SAM-e) as the methyl group donor (11) H3K27 methylation activity is dependent on incorporation into a complex with EED and SUZ12 (11) SET domain (SAM-e binding site) has been the target of the majority of EZH2 inhibitors	Loss-of-function mutations in the EED binding domain or catalytic SET domain can destabilize EZH2 and eliminate H3K27 methylation activity (86) Activating mutations of Y641, A677, A687 in the SET domain enhance H3K27me3 activity (87–90)
EED	WD-40 repeats	EED	WD-40 repeats form the aromatic cage that interacts with the histone methylation mark (11) EED binding to histone trimethyl mark allosterically activates EZH2 catalytic activity (34)	Mutations in the aromatic cage can completely eliminate recognition of the histone methylation mark (52)
SUZ12	C-terminalVEFS Box	SUZ12	Stabilizes PRC2 complex through interactions with EZH and EED (91)	
RBBP4/RbAp48 RBBP7/RbAp46	WD40 repeats	RBBP4 RBBP7	Involved in histone binding, particularly H4. Found in multiple chromatin binding complexes like CAF-1, HDAC, NuRD, NURF and PRC2 complex (92–94)	
PCL1/ PHF1 PCL2/ MTF2 PCL3/ PHF19	N-terminal TUDOR domain PHD-type zinc finger C-terminal Polycomb-like MTF2 factor 2 domain	PCL1 PCL2 PCL3	Binds to unmethylated CpG motifs, facilitating PRC2 recruitment to specific loci (24) Tudor domain binds to H3K36me, facilitating PRC2 recruitment and H3K27me3 deposition at those sites (95,96)	
AEBP2	Zinc finger	AEBP2	Boosts the histone methyl transferase (HMTase) activity of PRC2 on ubiquitinated H2A nucleosomes *in vitro* (97) Recognizes H2AK119ub1 to facilitate PRC2 recruitment (22) Acts as DNA targeting subunit (91,98) Mimics unmethylated H3K4 binding to RBBP4 which allosterically activates PRC2 activity (11)	AEBP2-null mESCs have been reported to show increased H3K27me3 levels due to formation of a hybrid PRC2 in these cells consisting of PRC2 core-JARID2-PCL2 (99) Two isoforms identified in human- an adult specific isoform (51 kDa) and an embryo-specific smaller isoform (32 kDa), both containing the zinc fingers (100)
JARID2	ARID DNA binding domain Jmj domain Zinc finger	JARID2	Boosts the HMTase activity of PRC2 *in vitro* (101) Recognizes H2AK119ub1 to facilitate PRC2 recruitment (22)	JARID2K116 can be methylated by PRC2 (JARID2K116me3) which can bind to EED and allosterically stimulate PRC2 enzymatic activity (102)
PALI1 PALI2	Helix-turn-helix DNA binding domain	PALI1	Vertebrate specific subunits, mutually exclusive in PRC2 with AEBP2 (103) PALI1 facilitate chromatin binding and promotes PRC2 enzymatic activity (104)	PRC2 methylates PALI1 at K1241 which allosterically activates PRC2 enzymatic activity through EED (104)
EPOP	BC box		Recruits Elongin BC to PRC2 sites (105)	
EZHIP/CATACOMB			Inhibitor of allosterically activated PRC2 H3K27me3 catalytic activity (106–108)	

## PcG PROTEINS IN CANCER

Tumor genome sequencing has revealed frequent alteration of genes encoding chromatin regulators, including PcG proteins (109). PcG proteins have traditionally been viewed as oncogenic due to their ability to prevent differentiation (110) and bypass senescence (111,112), in part by repressing the *INK*/*ARF* tumor suppressor genes (113,114). This function is important for maintaining proliferation of both normal stem cells and cancer stem cells (CSCs) (11,115). Despite a generally oncogenic function for PcGs, the role for individual subunits can vary tremendously, with some subunits acting as both an oncogene and a tumor suppressor depending on the context. Predicting which cancers are dependent on PcG function and then determining the best subunit to target is still challenging, although several trends have emerged based on clinical and experimental data.

### Genetic mutation of PcG genes in cancer

**Figure 3.**
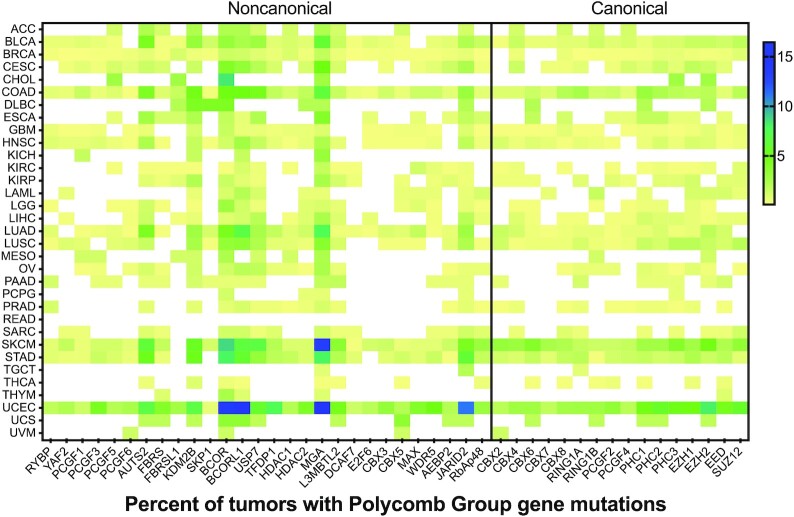
Heatmap of PcG subunit mutation frequency in cancer. The number of patient tumors with mutations in PcG genes was determined using TCGA datasets in cBioPortal, and the percentage in each cancer type is displayed as a heatmap. White boxes indicate that no mutational data was available.

From the TCGA mutation data, it is clear that some cancers have a high incidence of PcG gene mutation (Figure 3). The high mutation rates in many of these cancers, such as melanoma (SKCM), stomach cancer (STAD), colon cancer (COAD), uterine cancer (UCEC) and lung cancers (LUAD, LUSC) are more likely due to high mutational burden than any relevant function for PcG proteins (116). However, frequent mutations in some subunits, such as MGA and BCOR/BCORL1, as well as generally high mutation rates in cancers such as diffuse large B-cell lymphoma (DLBCL) and cholangiocarcinoma of the bile duct (CHOL) point to specific roles for Polycomb subunits in tumor suppression.

#### MGA

MGA mutations are frequently found in multiple cancer types (Figure 3). The oncogenic role of MGA mutation is likely not due to the loss of PRC1.6 function but instead due to the release of free MAX, which can associate with MYC, and facilitate MYC-mediated oncogenic transcription (117,118). Whether mutations in other PRC1.6 subunits affect MYC function in a similar manner has not been established.

#### BCOR/BCORL1

BCOR is the member of ncPRC1.1 that directly associates with BCL6 to facilitate repression at BCL6 binding sites. BCOR/BCORL1 mutations are found in an array of cancers (Figure 3), and inactivating BCOR mutations have been identified in various hematological, epithelial and central nervous system (CNS) neoplasms (119–123). Somatic mutations of *BCOR/BCORL1* are found in 10% acute myeloid leukemia patients (124–127), as well as in other hematological malignancies (128). BCOR is required for myeloid differentiation (129) and its deletion in mice disrupts H2AK119ub1-mediated repression of *Hox* genes during differentiation, leading to leukemia when paired with KRAS mutation (130).

#### DLBCL

Due to a prominent role for Polycomb in both B-cell and T-cell development, blood cancers have frequent genomic alterations in Polycomb genes (34,131). In most lymphoid malignant contexts, PRC subunits are overexpressed or activated; however, loss of function mutations are found in some specific types of lymphoid cancers, typically pediatric cancers, or cancers with mutations that activate Ras signaling (132).

#### TALL

Loss-of-function mutations in all three PRC2 subunits are found to be paired with Notch mutations in over 40% of early T-cell precursors, leading to T-cell acute lymphocytic leukemia (TALL) (133–138). Loss of PRC2 subunits in this cell type prevents T-cell differentiation by allowing repressed, but primed, promoters marked by H3K27me3 to become fully methylated and constitutively repressed (139). In this setting, it is not the repressive function of PRC2 that is important for tumor suppression, but the reversible nature of PRC2-mediated repression.

#### MPNST

In conjunction with additional tumor suppressors, the PRC2 subunits EED and SUZ12 (but not EZH2) are mutated in 85% of malignant peripheral nerve sheath tumors (MPNST) that develop in patients with NF1 mutations (140). These cancers have loss of H3K27me2 in addition to H3K27me3, indicating different mechanistic outcomes compared to EZH2 mutant cancers (141).

### Other genetic events leading to Polycomb loss-of-function

#### Oncohistone H3K27M

Lys27Met (K27M) mutations in histone H3.1 and H3.3 genes have been identified as genetic drivers in pediatric cancers (142–145). Even though mutations in a single copy of H3 constitute only a small fraction (3.63%–17.61%) of total H3 expressed, H3K27me3 levels are globally reduced (146–148) due to increased affinity to, and inhibition of, EZH2 by H3K27M (147). The inhibition is specific, however, to allosterically activated EZH2 involved in the spread of H3K27me3 marks, and does not affect EZH2 activity at CpG islands (108). Instead, increased H3K27me3 is observed at this small subset of sites (149) leading to efforts to inhibit EZH2 (150,151) in addition to targeting aberrant H3K27Ac-mediated gene activation (152,153).

#### EZHIP overexpression

Posterior fossa type A ependymomas have increased expression of EZHIP, which inhibits allosterically activated PRC2 through a motif similar to H3K27M (107,108,152,154). Similar to H3K27M cancers, a global decrease in H3K27me3 is observed in this cancer type; however, therapeutic strategies based on this mechanism have not yet been investigated.

### Genetic events leading to Polycomb dependency

Covered more extensively in (155).

#### EZH2 gain-of-function mutations

EZH2 is critical for the activation of resting B-cells and their entry into the cell cycle during germinal center (GC) development. As such, activation of PcGs through mutation or overexpression is commonly observed in B-cell leukemias and lymphomas. Heterozygous mutations of the Tyr641 (Y641F/N/S/H/C) are found in 22% of follicular lymphomas and GC type DLBCLs (87,156,157) and mutations of A677 and A687 have been described in isolated cases of DLBCLs (88). These mutants are inactive as monomethyltransferases but efficient at the di- and trimethylation, while wild-type EZH2 has the highest activity as a monomethyltransferase. The combined activities of WT and mutant EZH2 augments H3K27 trimethylation, increasing steady state levels of H3K27me3 and downstream gene repression (158). Because of the cooperative nature of WT and mutant EZH2, both forms are good drug targets and EZH2 inhibitors are effective against mutant DLBCL *in vivo* (158,159), leading to FDA approval of the EZH2 inhibitor tazemetostat for use in patients.

#### BCOR translocations

In contrast to BCOR’s role as a tumor suppressor in leukemia, activating BCOR alterations and translocations are common in pediatric sarcomas and CNS neuroectodermal tumors (CNS HGNET-BCOR), which have transcriptional profiles reminiscent of BCOR overexpression (160). One of the most common genomic alterations in BCOR is ITD (internal tandem duplication)–BCOR, which has duplications of the N-terminal PCGF1 binding domain. Similarly, translocations BCOR–CCNB3, BCOR–MAML3 and ZC3H7B–BCOR all include the PCGF1 binding domain, implicating a potential role for aberrant ncPRC1.1 function in oncogenesis. Whether BCOR-altered cancers are dependent on other ncPRC1.1 subunits remains to be determined.

#### Somatic mutation of UTX

UTX is a member of the MLL4 COMPASS complex and is the demethylase responsible for removal of H3K27me3 marks, among others (161). Inactivating mutations in *UTX* occur in several types of human cancer (162,163) and result in the loss of the JmjC domain essential for demethylase activity (161). Many *UTX* mutant cancers have increased levels of H3K27me3 and are more sensitive to EZH2 inhibitors (163).

#### SWI/SNF alterations

Similar to what is observed in *Drosophila*, Polycomb complexes and SWI/SNF chromatin remodelers are antagonistic in mammalian cells (164,165). As such, several cancers with SWI/SNF subunit alterations have increased H3K27me3 and an increased sensitivity to EZH2 deletion. In particular, pediatric rhabdoid tumors with loss of the SWI/SNF subunit SNF5 are dependent on EZH2 (166) and respond well to EZH2 inhibitors, which are now approved clinically for these cancers (167). Other SWI/SNF altered cancers that are dependent on EZH2 include SS18-SSX translocated synovial sarcoma (168), *ARID1A*-mutated ovarian cancer (169), *SMARCA4*-mutated lung (170) and ovarian cancers (171), and *PBRM1*-mutated renal cancers (172). Although the mechanism is not completely clear, some of the above cancers are dependent only on non-enzymatic functions of EZH2 (171).

#### MLL translocations

The fusion of the N-terminal portion of MLL with subunits of the super elongation complex, primarily ENL, AF9, or AF4, initiates leukemia through the constitutive activation of *HOX* and other hematopoietic stem cell maintenance genes. In MLL-rearranged leukemia, PRC2 promotes acute leukemogenesis through repression of general senescence regulators (173), and disruption of EED or both EZH paralogs inhibits growth of these leukemias (174).

In a more specific dependency, PRC1 subunit CBX8 interacts directly with AF9/ENL and is required for MLL-AF9 leukemogenesis in mice (67). Knockdown of *CBX8* or inhibition of the CBX8 chromodomain significantly reduces viability and *HOX* gene expression in MLL-AF9 leukemia cell lines (175). In this context, CBX8 contributes to gene repression via RING1B association, and contributes to gene activation via AF9/ENL association (65,176). An activating role for CBX8 may be a result of CBX8 incorporation into other complexes (67) or a result of selective CBX8 antagonism by MLL-AF9 (62,65). The exact biochemical role of CBX8 in MLL-AF9 leukemia remains to be elucidated.

### Cancers with general upregulation of Polycomb group genes

**Figure 4.**
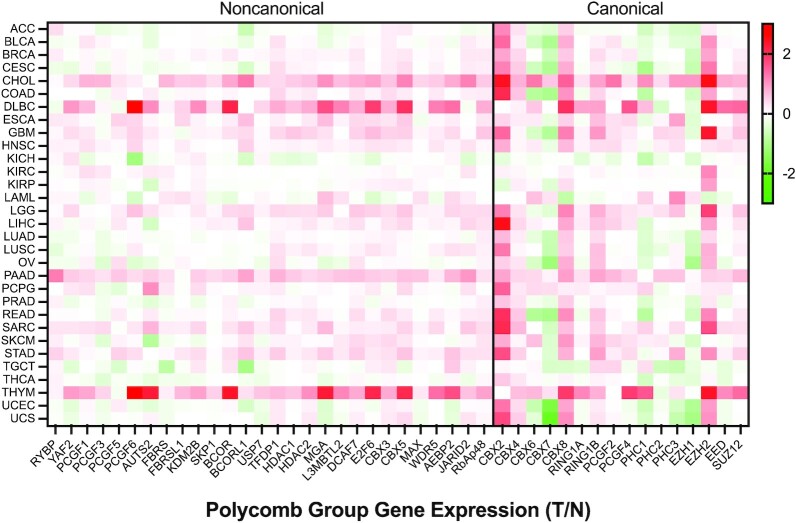
Heatmap of transcriptional changes in PcG subunits in cancer. Transcriptional fold change of both noncanonical and canonical Polycomb genes in TCGA tumor samples is normalized to TCGA normal, which is obtained from normal tissues near the tumors (T/N) using GEPIA2. Increases (red) or decreases (green) in cancer are displayed as Log2 fold change.

Comparing RNA-Seq data obtained from TCGA tumors to TCGA matched normal tissue, we defined differential gene regulation for all PcG genes across TCGA cancers (Figure 4). Several cancers have increased transcription of almost all PcG genes; these cancers include cholangiocarcinoma of the bile duct (CHOL), diffuse large B-cell lymphoma (DLBCL), pancreatic cancer (PAAD) and thymoma (THYM). The upregulation of Polycomb subunits has been confirmed in cholangiocarcinoma and pancreatic cancer (177,178) but has yet to be investigated in thymomas. While therapeutic targeting of PcG has not been extensively explored in any of these cancers, recent studies in pancreatic cancer indicate that targeting EZH2 or BMI1 may be effective in certain aggressive subtypes or cell populations (179,180).

**Figure 5.**
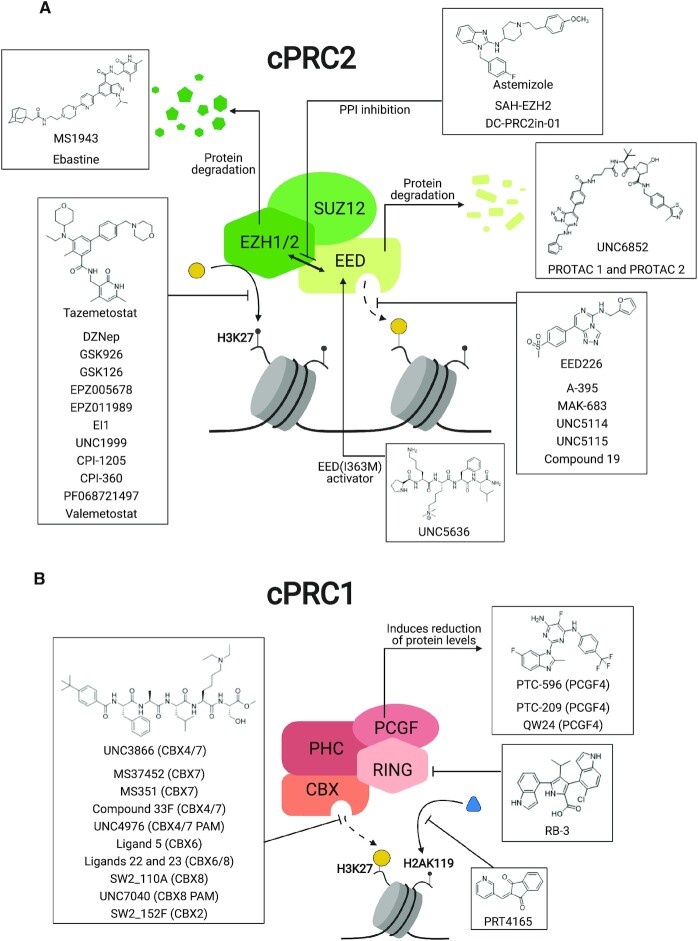
Mechanism of Action (MoA) for PcG protein modulators. Representative modulators targeting (**A**) PRC2 and (**B**) PRC1 are shown with illustrations of their MoA. PPI: Protein–protein interaction.

#### DLBCL

The oncogenic role of PcG upregulation in B-cell lymphoma is well-established and is related to a requirement for Polycomb in germinal center activation (see Section 2.1) (181). While EZH2 inhibitors are not as cytotoxic in DLBCL with increased EZH2 as they are in DLBCL with activating mutations in EZH2 (see Section 2.4), they still inhibit growth and may be effective in combination therapies (182).

BMI1 has been studied extensively in the context of leukemia, and was one of the first PcG proteins identified as oncogenic in hematological diseases (183,184). Ectopic expression of BMI1 gives rise to B- and T-cell lymphomas (185,186), and deletion of BMI1 delays primary leukemia and blocks secondary leukemia (187–189). These results implicate cPRC1 as a potential target; however, the best therapeutic strategy to modulate cPRC1 function in this setting is unclear.

The upregulation of ncPRC1 subunits is also important in DLBCL, particularly subunits of ncPRC1.1, which is important for silencing B-cell differentiation genes during the development of DLBCL (190). This is likely related to the mechanism by which ncPRC1.1 restricts myeloid differentiation by cooperating with BCL6 and PRC2 to repress myeloid regulator genes (129,191). BCL6 inhibitors and degraders are under development (192–194), and ncPRC1.1-specific inhibition may be similarly effective. KDM2B has two potentially targetable domains including the JmjC demethylase domain and the Zinc Finger (ZF)-C_XX_C DNA binding domain. Inhibitors targeting other JmjC domains have been reported; however, selectivity has been challenging (195). If resolved, selective inhibitors to either domain could be extended to the development of KDM2B-specific inhibitors or degraders for DLBCL treatment.

### PRC paralogs as oncogene/tumor suppressor pairs

While many cancer-specific alterations in PcG subunits are related to specific developmental pathways or cancer-specific phenotypes, some sets of paralogs are universally up and downregulated across multiple cancers, including EZH2/EZH1, RING1B/RING1A and (CBX2/CBX8)/(CBX6/CBX7) (Figure 4).

#### EZH

EZH2 upregulation paired with EZH1 downregulation in tumors is likely related to the fact that EZH2 is expressed only in actively dividing cells and plays a vital role in cellular proliferation, while EZH1 is expressed in both dividing and differentiated cells. While some cancers are dependent on both paralogs, others are dependent only on EZH2, leading to the development of both selective and nonselective catalytic inhibitors (196). In some cancers with EZH2 upregulation, inhibition of the catalytic activity alone is insufficient to stall tumor growth, implicating catalytic-independent roles for EZH2 in cancer progression. This may include non-enzymatic roles for EZH2 in gene activation (197), as well as non-transcriptional functions, such as in cytosolic actin polymerization during metastasis (198). To modulate catalytic-independent functions of EZH2, selective degraders of EZH2 may be more generally effective as a therapeutic strategy (199).

#### RING

RING1B is the more commonly upregulated RING paralog in cancer, and as the catalytic subunit of PRC1, represents a good therapeutic target (Figure 4); in contrast, RING1A is commonly downregulated in cancers and low expression correlates with poor prognosis, indicating that it may act as a tumor suppressor (200). Considering this, as well as the central importance of RING activity in many normal cells, the development of RING1B-specific inhibitors may prove more selective for cancer.

#### CBX

CBX paralogs are frequently misregulated in cancer, with CBX2 and CBX8 as the most commonly upregulated paralogs, and CBX7 and CBX6 as the most commonly downregulated (Figure 4). Supporting generally oncogenic roles, CBX2 and CBX8 have emerged as targets in lymphoma (190), hepatocellular carcinoma (201), breast cancer (202,203), prostate cancer (204) and ovarian cancer (205), while CBX6 and CBX7 have been implicated as tumor suppressors in thyroid cancer (206) lung cancer (207), glioblastoma (208), bladder cancer (209), cervical cancer (210) and breast cancer (211). There are exceptions, however; CBX7 acts as an oncogene in lymphoma and prostate cancer (212,213), and CBX6 acts as an oncogene in hepatocellular carcinoma (214). A correlation to any known role for CBX2 and CBX8 in development or cell cycle regulation is not apparent; however, the recently uncovered biochemical role for CBX2, and to a lesser degree for CBX8, in phase separation, may be related to oncogenic function (215,216). Whether selective inhibition of these paralogs has general utility for cancer therapy remains to be seen.

### Cancer subpopulations with dependencies on PcG proteins

In many cancers, PcG proteins are not involved in the development of tumors but are instead involved in cancer progression or resistance to therapy, either through facilitating therapy-resistant subtypes or therapy-resistant cell populations.

#### Metastatic prostate cancer

Multiple PcG proteins are potential targets in prostate cancer, including EZH2, CBX2 and BMI1 (217–223). The most established target is the PRC2 subunit EZH2, which is overexpressed in metastatic prostate cancer and is strongly correlated with cell cycle and DNA repair genes. EZH2’s role in prostate cancer may be at least partially separate from its function in PRC2, as it promotes androgen receptor (AR)-dependent gene activation and DNA damage repair in a manner independent of H3K27me3 (224,225). EZH2 is also upregulated in therapy-resistant neuroendocrine prostate cancer, where it functions in conjunction with CBX2 to repress AR target gene expression and promote resistance to AR-targeted therapies (226,227). EZH2 inhibition across a variety of therapy-resistant prostate cancers shows promising results, particularly to reverse chemotherapy and radiotherapy resistance (228,229). The oncogenic functions of RING1B and BMI1 along with the fusion BMI1-COMMD3 (230–233) may be additional targets for treating advanced prostate cancer.

#### Glioblastoma multiforme

Glioblastoma multiforme (GBM) is malignant Grade IV brain tumor, composed of heterogeneous cell populations, predominantly abnormal astrocytic cells. Within this population are glioma stem-like cell (GSC) with high expression of PcG genes (234). This population is responsible for resistance to radiation or chemotherapy and tumor repopulation after treatment (196,235,236). Short-term EZH2 depletion or inhibition stalls tumor growth, while prolonged EZH2 depletion drastically alters tumor cell identity and enhances tumor progression (196). The expression of other PcG subunits, such as BMI1, is also enriched in GBM cancer stem cells (237), although in a different GSC population, indicating that dual targeting of multiple PRC complexes may be necessary (196).

#### Colorectal cancer

Similar to glioblastoma, targeting cancer-initiating cells (CICs) in colorectal cancer is a promising strategy to prevent tumor recurrence after therapy. Human colorectal CIC function is dependent on BMI-1 (PCGF4), a subcomponent of PRC1 (238).

## POLYCOMB GROUP PROTEIN MODULATORS: INHIBITORS/ DEGRADERS/ ACTIVATORS

Considering the importance of PcG proteins in gene regulation and cancer progression, many chemical approaches have been developed to target different aspects of Polycomb function. An overview of the different subunits targeted and the strategies for modulation are described in Figure 5 and summarized with chemical structures in Table 2. Additional derivatives of similar scaffolds are not all included.

**Table 2.** Mechanism of action and structure of Polycomb modulators

Target subunit	Ligand	Mechanism of action	Structure	Reference	Associated Clinical Trials (NCT number)
**PRC2**					
EZH2	3-Deazane-planocin A	Inhibits the histone methyltransferase activity of EZH2 while inducing degradation of the PRC2 core subunits EZH2, EED, and SUZ12. In immunocompromised mice, this compound reduced the time of formation of tumors originating from prostate cancer cells	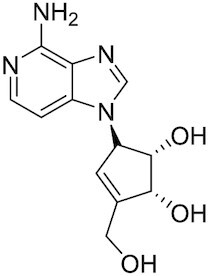	(240)	
EZH2	GSK926	SAM-e competitive inhibitor discovered from a high-throughput screening of the GSK compound collection. Reduces H3K27me3 levels in a breast cancer cell line and inhibits cell proliferation in breast and prostate cancer cell-based models	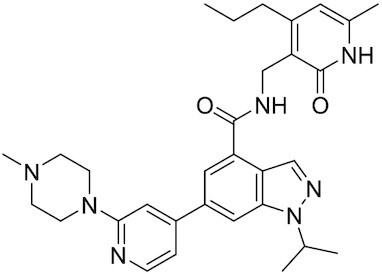	(241)	
EZH2	GSK126	In a similar fashion as GSK926, GSK126 was discovered from a high-throughput screening of the GSK compound collection. Highly selective for EZH2 over other methyltransferases. Inhibits cell proliferation in B-cell lymphoma cell-based and murine models that contain an EZH2-activating mutation	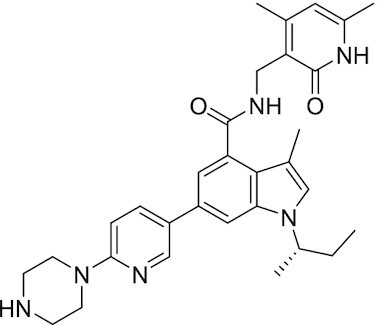	(159)	NCT02082977
EZH2	EPZ005678	Selectively reduces H3K27 methylation by EZH2 *in vitro* and in lymphoma cell-based models. Treatment of lymphoma cells bearing a mutant EZH2, leads to antiproliferative effects, indicating that these cancers are critically dependent on mutant EZH2	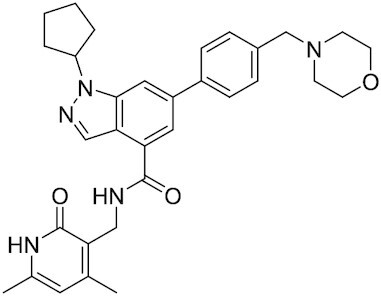	(158)	
EZH2	EPZ-6438 (tazemetostat)	Discovered along with EPZ005678 but shows better potency and oral bioavailability in animals. Treatment of mice bearing a lymphoma xenograft with mutant EZH2 reduces cell growth in a concentration dependent manner. FDA-approved for follicular lymphoma and epithelioid sarcoma with SNF5 deletions	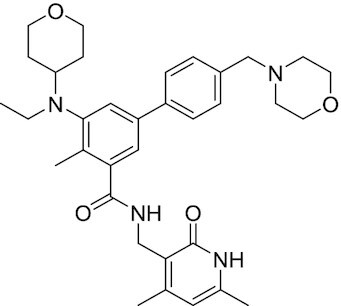	(243)	NCT03456726 NCT02601950 NCT01897571 NCT03213665 NCT02860286 NCT02601937 NCT04557956 NCT04762160 NCT03010982 NCT03874455 NCT02875548 NCT04179864 NCT03854474 NCT03028103 NCT04917042
EZH2	EPZ011989	Inhibits EZH2 in a mouse xenograft model of DLBCL, resulting in tumor growth inhibition while showing oral bioavailability	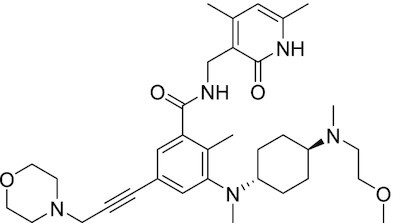	(247)	
EZH2	EI1	Inhibits the methyl-transferase activity of EZH2/PRC2 leading to reduction of H3K27 methylation over other H3 methylation marks. EI1 shows antiproliferative effects and down-regulates the proliferation gene signature in DLBCL	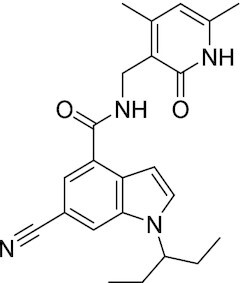	(248)	
EZH2	UNC1999	Inhibits the methyl-transferase activity of EZH2 and EZH1 by acting as competitor of SAM-e. UNC1999 is orally bioavailable and shows no adverse effects in Swiss albino mice	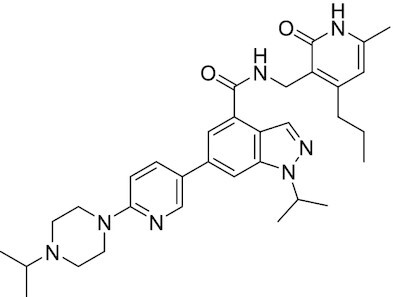	(249)	
EZH2	CPI-360	Competes with SAM-e for the EZH2 SET domain, leading to reduction of H3K27 trimethylation levels without affecting the protein levels of EZH2, SUZ12, and EED. CPI-360 has antiproliferative effects in different lymphoma cell-based models as well as in a human B-cell non-Hodgkin lymphoma murine model	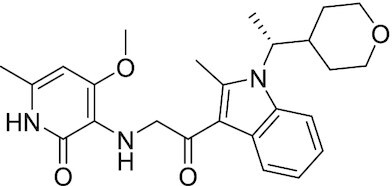	(251)	
EZH2	CPI-1205	Binds to the EZH2 catalytic domain. CPI-1205 proved to be efficacious, well-tolerated and highly bioavailable in a lymphoma xenograft model. Currently under clinical trials	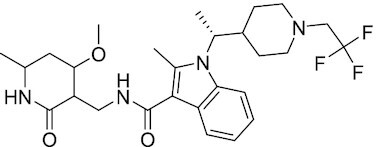	(291)	NCT02395601 NCT03480646 NCT03525795
EZH2	PF06821497	EZH2 catalytic inhibitor effective in mouse xenograft model of DLBCL	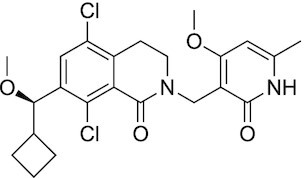	(253)	NCT03460977
EZH1/2	Valemetostat (DS-3201b)	EZH1/EZH2 dual inhibitor with activity in DLBCL, as well as AML, TAL and urogenital cancers	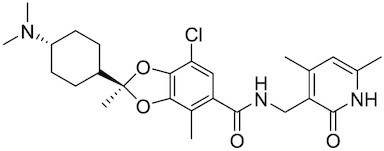	(255)	NCT04703192 NCT04842877 NCT04102150 NCT04388852
EZH2	MS1943	First-in-class EZH2 degrader, selective for EZH2 over other methyltransferases. Induces EZH2 degradation and cytotoxicity in triple-negative breast cancer cell-based models	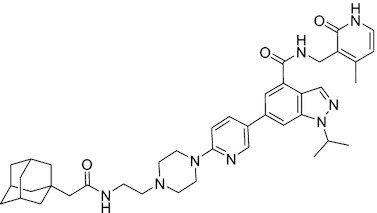	(199)	
EZH2	Ebastine	Initially discovered as an antihistamine drug, repurposed as an EZH2 inhibitor by decreasing EZH2 expression and reducing the levels of H3K27me3 in breast cancer and prostate cancer cells. Also active in a triple-negative breast cancer murine model	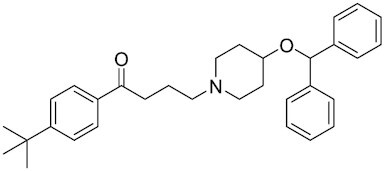	(229)	
EZH2-EED	Astemizole	Disrupts EZH2-EED protein-protein interaction, which results in inhibition of the methyltransferase activity of PRC2. Astemizole inhibits proliferation of DLBCL cells	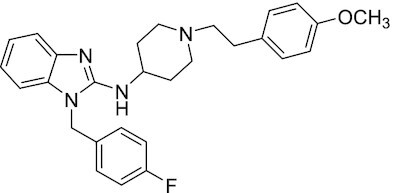	(258)	
EZH2-EED	SAH-EZH2	Peptidomimetic of stabilized alpha-helix of EZH2 which disrupt the EZH2-EED interaction leading to reduced H3K27me3 and EZH2 protein levels. SAH-EZH2 is capable of inducing growth arrest in leukemia cells and shows antiproliferative effects in B-cell lymphoma cell lines.	FSSNRQKILERTEILNQEWKQRRIQPV	(259)	
EZH2-EED	DC-PRC2in-01	Inhibits EZH2-EED interaction leading to reduced H3K27me3, as well as degradation of PRC2 core subunits. DC-PRC2in-01 inhibits PRC2-driven lymphomas cell growth and demonstrates cell cycle arrest at G0/G1 phase	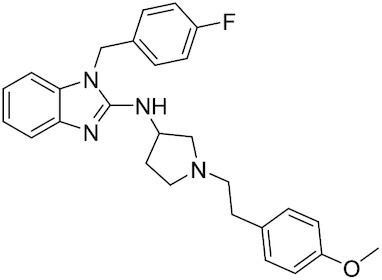	(260)	
EED	A-395	Inhibits EED H3K27me3 recognition by binding to the H3K27me3 binding pocket. Inhibits growth in DLBCL cell lines that have acquired resistance to EZH2 inhibitors. Also active in a xenograft murine model	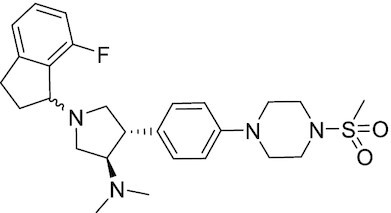	(292)	
EED	EED226	Binds to the EED binding pocket that recognizes H3K27me3. Reduces H3K27 methylation in a human B-cell non-Hodgkin lymphoma cell line and inhibits tumor proliferation in a human B-cell non-Hodgkin lymphoma murine model	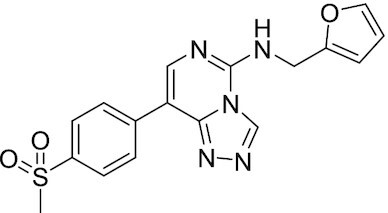	(263)	
EED	MAK-683	Currently under Phase I/II study to be evaluated as an anti-tumor agent in DLBCL, nasopharyngeal carcinoma (NPC) or other advanced solid tumors for whom no further effective standard treatment is available	Unknown	(265)	NCT02900651
EED	UNC5115	Discovered with UNC5114. Binds to the H3K27me3 binding pocket and inhibits the catalytic activity of PRC2	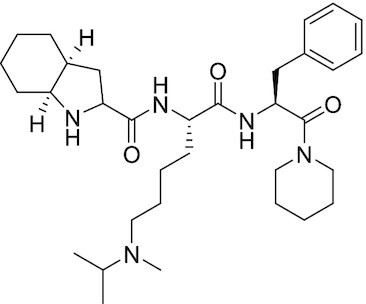	(266)	
EED	Compound 19	Acts as a competitor for the H3K27me3 binding pocket in EED, leading to reduction in the methyltransferase activity of PRC2. Inhibits growth in a DLBCL cell line.	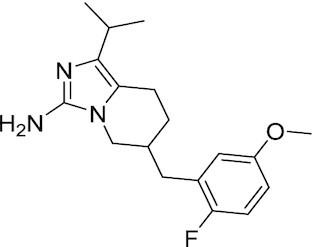	(267)	
EED	UNC6852	Bivalent chemical degrader that binds to EED and leads to degradation of PRC2. Derived from EED226 and a VHL ligand. Decreases H3K27me3 levels in DLBCL cell lines	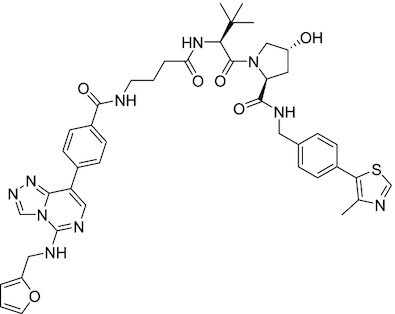	(268)	
EED	PROTAC 2	Degrades EED along with EZH2 and SUZ12. PROTAC 2 is a more potent degrader than its analogue PROTAC 1. Both molecules inhibit growth in a DLBCL cell line as well as a rhabdoid cancer cell line	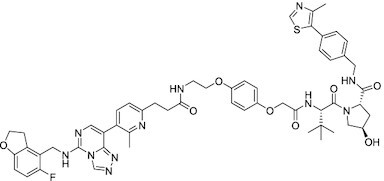	(269)	
EED	UNC5636	Peptidomimetic compound that selectively activates EED bearing a I363M mutation. This promotes PRC2 catalytic activity shown by the incorporation of a methyl group to lysine 27 of H3 peptide	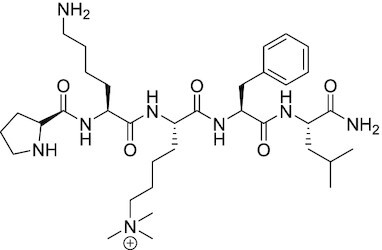	(270)	
**PRC1**					
RING	PRT4165	Inhibits H2A ubiquitination of topoisomerase Top2α at double-strand break sites in cells	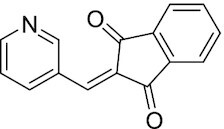	(275)	
RING	RB-3	Binds RING1B and alters protein conformation, preventing association with histones and subsequent H2A119Ub	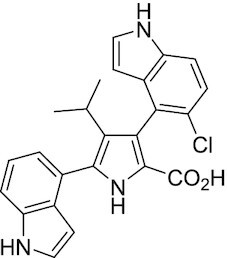	(276)	
PCGF4 (BMI-1)	PTC-209	Inhibits colorectal cancer-initiating cells by reducing the protein levels of PCGF4 (BMI-1)	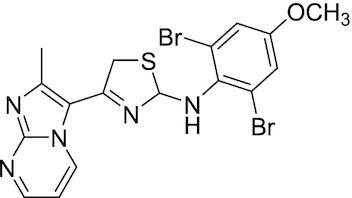	(238)	
PCGF4 (BMI-1)	PTC-596	Reduces the levels of functional BMI-1 by inducing its hyper-phosphorylation. Currently under Phase 1 clinical trials	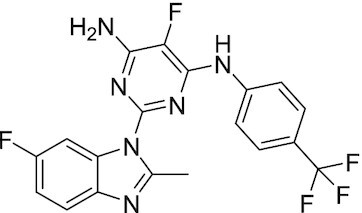	(277)	NCT03206645 NCT03761095 NCT03605550 NCT02404480
PCGF4 (BMI-1)	QW24	Induces BMI-1 protein degradation through the autophagy-lysosome pathway, leading to inhibition of colorectal CICs’ self-renewal	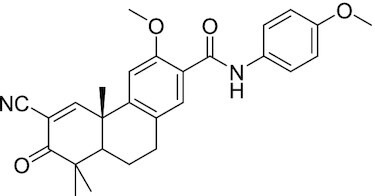	(278)	
CBX4/7	UNC3866	Binds to the ChD of the CBX paralogs, preventing them from binding methyllysine. UNC3866 inhibits proliferation of PC3 prostate cancer cells	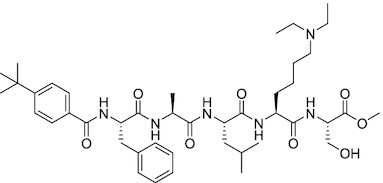	(282)	
CBX7	MS37452	Displaces CBX7 from the *INK4A/ARF* locus in prostate cancer cells, hence de-repressing the transcription of *p16/CDKN2A*	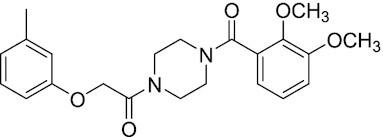	(283)	
CBX7	MS351	Discovered via structure-guided drug design, MS351 inhibits CBX7 binding to H3K27me3 when it is bound to RNA. It also derepresses CBX7 target genes in both mouse embryonic stem cells and PC3 prostate cancer cells	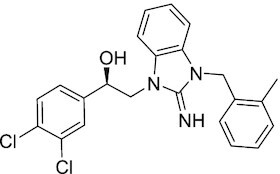	(284)	
CBX7	Compound 33F	Developed using rational design to modify a L3MBTL1 methyllysine binding inhibitor	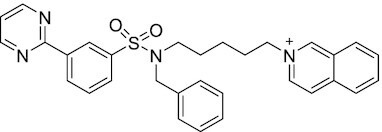	(285)	
CBX4/7	UNC4976	Allosteric modulator of CBX7, abrogating its function as reader of H3K27me3 marks and increasing its non-specific binding to DNA	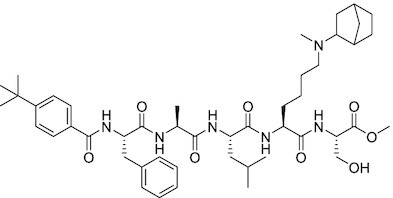	(286)	
CBX6	Ligand 5	Binds to the beta groove of CBX6, which includes the lysine trimethylation binding pocket along with a (−2) pocket and a hydrophobic cleft extending from the binding site	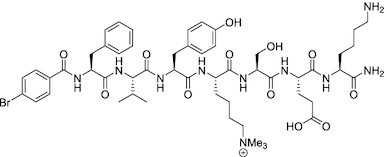	(287)	
CBX6/8	Ligand 22	Selectively binds to both CBX6 and CBX8 over other CBX ChDs	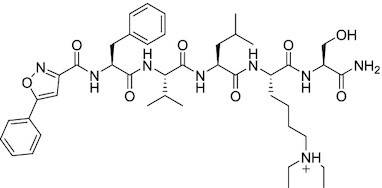	(288)	
CBX8	SW2_110A	Binds to the ChD of CBX8 and prevents its association with chromatin, leading to inhibition of proliferation and deactivation of MLL-AF9 target genes in THP1 leukemia cells	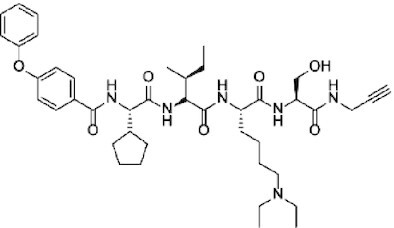	(175)	
CBX8	UNC7040	Allosteric modulator of CBX8, abrogating its function as reader of H3K27me3 marks and increasing its non-specific binding to DNA, leading to inhibition of proliferation in lymphoma cells	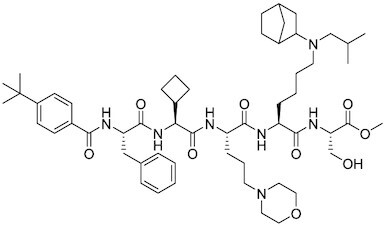	(290)	
CBX2	SW2_152F	Selective CBX2 chromodomain inhibitor. Prevents and reverts neuroendocrine differentiation in prostate cancer cells	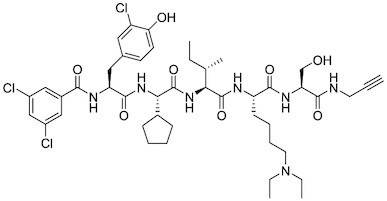	(226)	

### PRC2 modulators

Overview of this section is depicted in Figure 5A.

#### EZH2 catalytic inhibitors

Given the central role for H3K27me3 in Polycomb-mediated repression, most inhibitors to date target the catalytic activity of EZH2. Within this category, most developed inhibitors bind to the SET domain of EZH2 and compete with S-adenosyl-L-methionine (SAM-e) cofactor binding. EZH2 enzymatic inhibitors have impressive efficacy in certain preclinical and clinical cancer models. A more detailed description of the medicinal chemistry is highlighted in (239).

##### 3-Deazaneplanocin A (DZNep)

DZNep is an inhibitor of the enzyme S-adenosylhomocysteine hydrolase. Inhibition of this hydrolase results in a reduction of cellular SAM-e, thereby inhibiting all SAM-e-dependent enzymes, including EZH2. DZNep effectively decreases the expression of PRC2 proteins EZH2, SUZ12, and EED, as well as global H3K27me3 levels, leading to the reactivation of the PRC2-repressed genes and apoptosis in breast cancer cell line MCF-7 and colorectal cancer cell line HCT116, but not in normal cells (240). However, the lack of specificity for EZH2 inhibition makes DZNep unsuitable as a probe of EZH2 dependency, leading to further efforts to elucidate its contribution to EZH2-independent processes.

##### GSK926 and GSK126

GSK926 was the first highly potent, SAM-e competitive and selective EZH2 inhibitor developed by GSK (241). GSK126 has improved properties and activity against cell migration and angiogenesis (159).

##### EPZ005687

EPZ005687 is a selective inhibitor of EZH2 (K_i_ = 24 nM) and therefore blocks H3K27 methylation (158). EPZ005687 displays 50-fold selectivity for EZH2 over the closest methyltransferase EZH1, and >500-fold selectivity over 15 other methyltransferases. EPZ005687 inhibits the growth of EZH2-activating-mutant lymphoma cells, with lesser effect on wild-type lymphoma, making it the first-in-class chemical probe for evaluating the effect of inhibiting EZH2 enzymatic activity in cancer. EPZ005687 is also effective in inhibiting the proliferation and migration of synovial sarcoma cells driven by a translocation in SWI/SNF subunit SS18 (242).

##### EPZ-6438 (Tazemetostat)

EPZ-6438 is a potent (*K*_i_ = 2.5 nM) and selective (35-fold over EZH1 and 4500-fold to other HMTs) SAM-e competitive small molecule inhibitor of EZH2. Although EPZ-6438 has similar MoA and selectivity in comparison to EPZ005678, EPZ-6438 has superior potency and drug-like properties. EPZ-6438 treatment of mice bearing EZH2-mutant non-Hodgkin lymphoma xenografts demonstrated complete and sustained tumor regressions with concurrent diminution of H3K27me3 levels in tumors. EPZ-6438 is currently FDA-approved for follicular lymphoma, B-cell non-Hodgkin lymphoma and epithelioid sarcoma (243–246).

##### EPZ011989

Optimized from EPZ-6438, EPZ011989 has a lower amine pK_a_, while the selectivity for EZH2 and potency against EZH2-mutant lymphoma are maintained (*K*_i_ < 3 nM). In addition, it reduces H3K27me3 levels in tumors and exhibits good bioavailability, metabolic stability, PK/PD profile, and *in vivo* activity (247).

##### EI1

The SAM-e competitive EZH2 inhibitor, EI1, was identified from a high-throughput screening (248). EI1 demonstrates potent inhibition of the enzymatic activity of both wildtype EZH2 and Y641F mutant with IC_50_ of 15 nM and 13 nM, respectively. DLBCL cells treated with EI1 exhibit genome-wide loss of H3K27 methylation and activation of PRC2 target genes, leading to decreased proliferation, increased cell cycle arrest, and apoptosis.

##### UNC1999

UNC1999 is an orally available and selective SAM-e competitive inhibitor with high potency for both EZH2 and EZH1 (249). UNC1999 suppresses global H3K27 trimethylation/dimethylation with concurrent gain of H3K27ac, leading to growth inhibition of mixed-lineage leukemia (MLL) cells (250). UNC1999 displays *in vivo* activity in a well-defined murine MLL-AF9 leukemia model, delaying MLL-AF9-induced leukemogenicity and prolonging survival (250).

##### CPI-1205 and CPI-360

CPI-360 is a highly potent (*K*_i_ = 0.5 nM) EZH2 inhibitor developed at Constellation Pharmaceuticals from a pyridone-based high-throughput screen hit. It displays activity in both mutant EZH2-containing lymphomas as well as lymphomas with WT EZH2 (251). Optimized compound CPI-1205 is effective *in vivo* and well-tolerated in clinical trials (252). Further modifications to improve residence time and metabolism are being pursued.

##### PF068721497

A catalytic inhibitor developed at Pfizer from a focused library of dimethylpyridones. It is potent against both WT and mutant EZH2 and induces tumor regression in DLBCL mouse xenograft models (253).

##### Valemostat

A catalytic inhibitor of both EZH1 and EZH2 developed by Daiichi Sankyo Co. (254) It is active against EZH2-mutant DLBCL as well as AML (255). It is in clinical trials for these cancers, as well as for TALL and urogenital cancers.

#### EZH2 degraders and downregulators

##### MS1943

Although EZH2 inhibitors are effective in treating sarcoma and lymphoma, other cancers that are dependent on EZH2, such as triple negative breast cancer (TNBC), do not respond to catalytic inhibition. For these cancers, chemical approaches to degrade EZH2 are being explored. MS1943 is the first-in-class EZH2-selective degrader that effectively reduces EZH2 levels in cells (199). It is a bivalent compound consisting of an EZH2 inhibitor linked to an adamantane hydrophobic tag. The hydrophobic tag interacts with greasy core residues of the protein and destabilizes the protein folding, resulting in a partially folded protein that is subsequently degraded by the proteasome (256,257). MS1943 has profound cytotoxic effect in multiple TNBC cells *in vitro* and *in vivo*, with minimal effect on normal cells.

##### Ebastine

Ebastine, a marketed antihistamine drug, reduces *EZH2* transcript levels, leading to decreased H3K27me3 levels in breast and prostate cancer cell lines. It decreases cancer cell growth and reduces tumor growth and progression in xenograft mice models (229). Ebastine demonstrates promise as an anticancer medication considering its potency as EZH2 inhibitor and its safety as an antihistamine that is already available on the market.

#### EZH2-EED interaction disruptors

Since EZH2 enzymatic function depends on its interaction with EED, disruption of this interaction provides another route to modulate EZH2 activity. Disruption of EZH2-EED interaction also reduces the protein levels of EZH2 and may have broader therapeutic implications for PRC2-addicted cancers that are either dependent or independent of its HMT activity (172,199).

##### Astemizole

This antihistamine drug has been recently identified as a small molecule inhibitor of EZH2-EED interaction. It inhibits PRC2 activity by depleting the PRC2 subunits and decreasing H3K27me3 levels, resulting in anti-proliferation activity in lymphomas (258).

##### SAH-EZH2

SAH-EZH2 is a stabilized alpha-helix mimetic of the EZH2 peptide. It selectively inhibits H3K27 trimethylation of PRC2 *in vitro* and selectively suppresses the growth of leukemia and lymphoma cells by disrupting the EZH2-EED interaction (259). Stapled peptides have sub-optimal pharmacokinetics, reducing their utility as single dose agents; however, they may have therapeutic potential administered along with enzymatic inhibitors to boost efficacy and decrease resistance.

##### DC-PRC2in-01

The co-crystal structure of astemizole in complex with EED was used for the structure-guided design of DC-PRC2in-01, a novel EZH2-EED interaction inhibitor (260). With a *K*_d_ of 4.56 μM, it causes degradation of PRC2 core subunits and a decrease in global H3K27me3 levels in lymphoma cancer cells.

#### EED inhibitors

The PRC2 subunit EED binds to the H3K27me3 mark and augments EZH2 enzymatic activity. As an alternative strategy to inhibiting the PRC2 methylation function, targeting EED can reduce PRC2 activity and prevent PRC2 binding to histones. EED inhibitors may also be advantageous in being capable of targeting both wildtype and mutant EZH2-dependent functions.

##### A-395

A-395 binds to the WD40 domain of EED, competing with H3K27me3 in the aromatic binding pocket with an IC_50_ of 7 nM. Inhibition of H3K27me3 binding prevents allosteric activation of PRC2; this resembles the results achieved with EZH2 inhibitors, but expands with activity against cell lines with acquired resistance to EZH2 inhibitors (261). Further structure-activity relationship studies on this compound found that 2,6-disubstitution of the N-benzyl improved the binding and cellular activities (262).

##### EED226

EED226 is a potent and selective PRC2 inhibitor developed from a high throughput screen hit that directly binds to the WD40 domain of EED. EED226 binding to EED induces a conformational change in the aromatic cage leading to the destabilization of EED-H3K27me3 interaction and a loss of PRC2 enzymatic activity. EED226 is effective in treating cancers susceptible to EZH2 inhibition, as well as cancers with a mutant EZH2 protein resistant to SAM-e competitive inhibitors (263). Several additional small molecules (EED666, EED162, EED210, and EED709) bind to EED in a similar manner, defining a common mechanism of inhibition and providing a starting point for further inhibitor development (263,264).

##### MAK683

MAK683 is an allosteric inhibitor of PRC2 developed by Novartis (265). Similar to EED226, it binds EED and alters the conformation of the EED-H3K27me3 binding pocket, preventing EED-H3K27me3 interaction and EZH2 activation. This drug is being tested in clinical trials for patients with DLBCL, nasopharyngeal carcinoma, gastric cancer, ovarian cancer, prostate cancer and sarcomas.

##### Peptidomimetic ligand UNC5114 and UNC5115

Using JARID2-K116me3 peptides as a starting point, smaller, more potent peptidomimetic ligands for the EED aromatic cage were developed. UNC5114 (*K*_d_ = 0.68 ± 0.05 μM) and UNC5115 (*K*_d_ = 1.14 ± 0.14 μM) exhibit 10-fold improvement in affinity and physicochemical properties compared with JARID2-K116me3 (114–118,266) and inhibit allosteric activation of PRC2 catalytic activity by EED.

##### Compound 19

A set of fragment-sized small molecules (compounds 14, 16, and 19) were discovered using structure-guided inhibitor design. The optimized compound 19 inhibits PRC2 with IC_50_ = 1.3 μM and inhibits rhabdoid cell growth with IC_50_ = 2.9 μM (267).

#### EED proteolysis targeting chimeras (PROTACs)

##### UNC6852

An EED-targeted bivalent chemical degrader UNC6852 selectively degrades EED and other PRC2 components through recruitment of the VHL ubiquitin ligase. UNC6852 inhibits PRC2 catalytic activity, decreases H3K27me3 levels, and inhibits proliferation of DLBCL cell lines. PRC2-targeted degraders have the potential to overcome acquired resistance to EZH2 inhibitors. More importantly, UNC6852 provides a useful tool for identifying and treating cancers dependent on non-catalytic functions for PRC2 (268).

##### PROTAC 1 and PROTAC 2

Simultaneously to UNC6852, a team at AstraZeneca reported different EED-based PROTAC molecules that utilize VHL recruitment to degrade PRC2 subunits and reduce PRC2 activity (269).

#### EED activators

##### UNC5636

Loss-of-function (LOFs) mutations in cancer, unlike gain-of-function mutations, are bigger challenges for targeted therapeutics. A subset of myeloid disorders has a I363M LOF mutation of EED that leads to a loss in EZH2 enzymatic activity. UNC5636 was developed using computational simulations and structure-based design to selectively stimulate the catalytic activity of PRC2-EED-I363M and not WT PRC2. Although the compound lacks cell permeability, this work demonstrates the feasibility of developing targeted agonists for reversing the LOF mutant phenotype (270), which can also be a promising direction for other PcG LOF mutations.

#### Limitations of PRC2 inhibitors

Although EZH2 demonstrates a remarkable level of substrate specificity in catalyzing methylation of only one lysine (H3K27) among all histone peptide substrates, various *in vitro* and cell-based studies indicate that EZH2 has additional substrates such as JARID2, H2BK120, talin, and transcription factors such as STAT3, which may or may not be desirable targets for cancer (246,271–274). In addition, the cytotoxic effects of EZH2 inhibition on adult stem cells remain a major concern in therapeutical strategies.

### PRC1 inhibitors

An overview of this section is summarized in Figure 5B.

RING inhibitors

#### PRT4165

PRT4165 inhibits RING1A and RING1B E3 ubiquitin ligase activity *in vitro*. While inhibition of PRC1-mediated H2A monoubiquitination in cells has not been shown, it does inhibit polyubiquitination at double stranded breaks, which is proposed to be downstream of PRC1 activity (275). This molecule is the first chemical inhibitor of PRC1 E3 ubiquitin ligase activity, although its low potency and structural similarity to pan assay interference compounds raises concerns over cellular activity and selectivity.

#### RB-3

Fragment-based screening and subsequent optimization of molecules that bind to the BMI1/RING1B dimer recently produced the first selective inhibitor of PRC1 RING ubiquitin ligase activity (276). This inhibitor binds RING1B with 3.6 μM affinity and alters the conformation to block ubiquitination activity. It is selective for PRC1 and effectively blocks H2AK119ub1 in cells, but it is not selective for RING1B over RING1A or BMI1 over PCGF1, indicating that it likely blocks all PRC1-mediated ubiquitination. While complete inhibition of PRC1 may be toxic to some cell types, the reduction of PRC1 ubiquitin ligase activity, or subsequent development of inhibitors selective for individual PRC1 subcomplexes, may prove therapeutically useful.

#### BMI-1 inhibitors

##### PTC209 and PTC596

PTC209 induces BMI-1 degradation and reduces PRC1 activity. Treatment of primary colorectal cancer xenografts with PTC-209 resulted in loss of colorectal CICs with irreversible tumor growth impairment and little cytotoxicity (238). Additional efforts from PTC Therapeutics resulted in PTC596, an orally bioavailable molecule currently in clinical trials (NCT02404480) for glioblastoma and fibrosarcoma. PTC596 inhibits APC/C^CDC20^ activity resulting in the persistent activation of CDK1 and CDK2, leading to hyperphosphorylation of BMI-1 and its degradation (277).

##### QW24

BMI-1 inhibitor QW24 decreases BMI-1 protein expression through the autophagy-lysosomal degradation pathway. Based on cell viability and protein regulation, QW24 is more potent than previously reported BMI-1 inhibitor PTC209. QW24 significantly inhibits stem-like properties in colorectal cancer cell lines, leading to attenuation of proliferation and metastasis. In animal studies, QW24 exhibits little toxicity in the subcutaneous xenograft model, with reduced tumor metastasis and increased mice survival (278).

#### CBX inhibitors

Five CBX proteins are incorporated into PRC1, which bind H3K27me3 via their chromodomains (ChDs). The flexibility of the ChD and high homology among the CBX ChD provide as major obstacles for developing potent, selective, and cell permeable CBX inhibitors.

##### CBX4/7

###### Peptidic inhibitors

CBX7 promotes proliferation of prostate cancer cells (279) and malignant hematopoietic progenitor cells (280). The first CBX ChD ligands were peptidic in nature with the highest affinity for CBX7, with 0.28 ±0.05 μM, and five-fold selectivity over CBX8 (281). UNC3866 was then developed with similar specificity for CBX7 and CBX4, but higher affinity and, most importantly, utilized a modification of the trimethyllysine to diethyllysine to allow for cell permeability. UNC3866 induces a senescence-like phenotype in PC3 prostate cancer cells and inhibits their proliferation at IC_50_ = 7.6 μM (282).

###### Small molecule inhibitors MS37452 and MS351

MS37452 and MS351 are small molecule CBX ChD inhibitors identified using high-throughput screening. Although the affinity to CBX7 ChD is low, they are selective for CBX7 over the other paralogs. Both molecules inhibit H3K27me3 binding; however, MS351 only inhibits H3K27me3 binding when CBX7 is bound to RNA (283,284). In PC3 prostate cancer cells, MS37452 and MS351 can inhibit CBX7 binding at the *INK4A/ARF* locus to induce transcriptional de-repression of *p16*/*CDKN2A* and inhibit PC3 growth.

###### Compound 33F

A set of small molecule inhibitors with low potency developed by rational adaption of inhibitors of L3MBTL1, a methyllysine-binding protein (285). This work identified multiple small-molecule inhibitors with modest to low potency (IC_50_: 257–500 μM).

###### Positive allosteric modulator UNC4976

UNC4976 is a modified version of UNC3866 with a large norcamphor group on the lysine instead of diethyls. The bulky modification does not change *in vitro* binding properties but does induce a conformational change that increases the affinity of CBX7 ChD to DNA/RNA. The enhanced efficacy of UNC4976 results from simultaneously antagonizing H3K27me3-specific binding while increasing non-specific DNA and RNA binding, shifting the equilibrium of CBX7-containing PRC1 from H3K27me3 target regions (286).

##### CBX6

###### Peptidic ligand 5

This inhibitor selectively inhibits the CBX6 ChD by occupying a small hydrophobic pocket adjacent to the aromatic cage. It displays good affinity (*K*_d_ = 900 nM) with *in vitro* selectivity >5-fold over other CBX paralogs (287).

##### CBX6/CBX8

###### Peptidic ligands 22 and 23

The first dual-selective inhibitors for CBX6 and CBX8 demonstrate high affinity to both CBX6 and CBX8 (288). Lead compounds from this study demonstrated efficacy in rhabdoid tumor cell line.

##### CBX8

###### Peptidic inhibitor SW2_110A

The selection of directed DNA-encoded libraries (DELs) against multiple ChDs led to the development of SW2_110A, a selective, cell-permeable inhibitor of the CBX8 ChD with a *K*_d_ of ∼800 nM, and minimal 5-fold selectivity (no binding to CBX4 and CBX6, 20-fold over CBX7, 5-fold over CBX2) for CBX8 ChD over all other CBX paralogs *in vitro*. SW2_110A specifically inhibits the association of CBX8 with chromatin in cells and inhibits the proliferation of THP1 leukemia cells driven by the MLL-AF9 translocation (175,289).

###### Allosteric modulator UNC7040

A potent positive allosteric modulator of CBX8, UNC7040, can antagonize H3K27me3 binding to CBX8 while increasing interactions with nucleic acids and participation in variant PRC1 (290). It has increased potency against CBX8 binding in DLBCL cell compared to similar inhibitors that only inhibit H3K27me3 binding.

##### CBX2

###### Peptidic inhibitor SW2_152F

The CBX2 ChD inhibitor SW2_152F has a *K*_d_ of ∼80 nM, and 24 to 1000-fold selectivity for CBX2 over the other CBX paralogs *in vitro*. This inhibitor blocks CBX2-facilitated neuroendocrine differentiation in prostate cancer, mainly through de-repressing AR signaling in neuroendocrine differentiated prostate cancer cells (226).

## CONCLUSIONS AND FUTURE OPPORTUNITIES

There are many cancers dependent on PcG proteins for initiation, progression, and chemotherapy resistance; however, the most effective method for targeting Polycomb function in a specific cancer is not always clear. Due to a central role for H3K27me3 in Polycomb-mediated repression and the relative homogeneity of PRC2 subunit composition, the majority of inhibitors developed to date target PRC2-mediated H3K27 trimethylation. Targeting the enzymatic SET domain of EZH1/2 is the most straightforward approach; however, drug-resistant mutant cancers, as well as cancers dependent on non-enzymatic PRC2 function, have necessitated alternate approaches to inhibit PRC2. Allosteric EED inhibitors, PRC2 disrupters, and EZH2/EED degraders can inhibit the growth of cancers insensitive to EZH2 enzymatic inhibitors, although the full utility of these compounds remains to be explored. Additional investigation of how this class of inhibitors affects both normal cells and cancers will reveal additional therapeutic utility.

Targeting PRC2 is effective against many Polycomb-dependent cancers; however, the complete loss of H3K27me3 may be detrimental to many normal cells, particularly adult stem cells. In addition, it is likely that not all PRC2 function in a particular cancer is oncogenic, and some PRC2 activity may actually be tumor suppressive, as has been shown in certain cancer types. Therefore, inhibiting a subset of Polycomb targets or inhibiting cancer-specific PRCs may be more effective in some situations. For instance, in many cancers CBX2/CBX8 are oncogenic while CBX6/CBX7 are tumor suppressive. Inhibitors for cPRC1, specifically CBX ChD inhibitors, can define the utility of more specifically targeting the subset of oncogenic cPRC1s that act downstream of PRC2. Current CBX ChD inhibitors are limited by poor pharmacological properties; however, they are useful for defining individual CBX paralog(s) to focus on as drug targets. The development of more potent CBX inhibitors will potentially require novel strategies, such as allosteric modulation or even degradation.

Targeting ncPRC1s is another strategy that may be more selective for certain cancers. ncPRC1 has higher ubiquitin ligase activity than cPRC1; however, if nonspecific RING enzymatic inhibitors prove toxic, a better strategy may be to target specific ncPRC1 variant subunits. ncPRC1.1 and ncPRC1.6 bind different regions of the genome but are functionally redundant in ESCs. In contrast, ncPRC1.1 is essential in B-cell lymphomas, while ncPRC1.6 acts as a tumor suppressor. Degraders provide an opportunity to target subcomplex-specific ncPRC1 subunits containing non-essential enzymatic or binding domains; for instance, targeting ncPRC1.1 through KDM2B or targeting ncPRC1.6 through L3MBTL2.

The functional redundancy for many Polycomb subunits along with the cell-type specific expression of PcG genes indicates that dual inhibition of PRC subunits may be a more effective pharmacological strategy. In probe development, selectivity is the highest priority; however, for drug development targeting of multiple functionally redundant PcG targets might be more effective in increasing cancer death and decreasing resistance. Even so, identifying and inhibiting just the subset of PcG targets important in cancer would reduce toxicity to normal cells. Defining the redundant and specific biological functions for individual PcG proteins in cancer is required for this polypharmacology approach; however, using genetic screens for multiple targets is highly challenging. Instead, small molecule inhibitors can be rapidly screened in combination to identify dual targeting approaches that selectively inhibit Polycomb activity in a cancer-specific manner. Increasing the diversity of Polycomb subunit inhibitors will significantly facilitate this approach. An increased understanding of how specific PRCs/subunits biochemically contribute to Polycomb activity will help with prioritizing targets, as well as developing effective strategies for inhibition.

## References

[1] Kennison J.A. The polycomb and trithorax group proteins of Drosophila: Trans-regulators of homeotic gene function. Annu. Rev. Genet. 1995; 29:289–303. 8825476 10.1146/annurev.ge.29.120195.001445

[2] Morey L., Santanach A., Blanco E., Aloia L., Nora E.P., Bruneau B.G., Di Croce L. Polycomb regulates mesoderm cell fate-specification in embryonic stem cells through activation and repression mechanisms. Cell Stem Cell. 2015; 17:300–315. 26340528 10.1016/j.stem.2015.08.009

[3] Müller J., Verrijzer P. Biochemical mechanisms of gene regulation by polycomb group protein complexes. Curr. Opin. Genet. Dev. 2009; 19:150–158. 19345089 10.1016/j.gde.2009.03.001

[4] Hauri S., Comoglio F., Seimiya M., Gerstung M., Glatter T., Hansen K., Aebersold R., Paro R., Gstaiger M., Beisel C. A High-Density map for navigating the human polycomb complexome. Cell Rep. 2016; 17:583–595. 27705803 10.1016/j.celrep.2016.08.096

[5] Schuettengruber B., Bourbon H.M., Di Croce L., Cavalli G. Genome regulation by polycomb and trithorax: 70 Years and Counting. Cell. 2017; 171:34–57. 28938122 10.1016/j.cell.2017.08.002

[6] Piunti A., Shilatifard A. The roles of Polycomb repressive complexes in mammalian development and cancer. Nat. Rev. Mol. Cell Biol. 2021; 22:326–345. 33723438 10.1038/s41580-021-00341-1

[7] Gao Z., Zhang J., Bonasio R., Strino F., Sawai A., Parisi F., Kluger Y., Reinberg D. PCGF Homologs, CBX Proteins, and RYBP Define Functionally Distinct PRC1 Family Complexes. Mol. Cell. 2012; 45:344–356. 22325352 10.1016/j.molcel.2012.01.002 PMC3293217

[8] Tavares L., Dimitrova E., Oxley D., Webster J., Poot R., Demmers J., Bezstarosti K., Taylor S., Ura H., Koide H. et al. RYBP-PRC1 complexes mediate H2A ubiquitylation at polycomb target sites independently of PRC2 and H3K27me3. Cell. 2012; 148:664–678. 22325148 10.1016/j.cell.2011.12.029 PMC3281992

[9] Jiao L., Liu X. Structural basis of histone H3K27 trimethylation by an active polycomb repressive complex 2. Science. 2015; 350:aac4383. 26472914 10.1126/science.aac4383 PMC5220110

[10] Mierlo G., Veenstra G.J.C., Vermeulen M., Marks H. The Complexity of PRC2 Subcomplexes. Trends Cell Biol. 2019; 29:660–671. 31178244 10.1016/j.tcb.2019.05.004

[11] Ciferri C., Lander G.C., Maiolica A., Herzog F., Aebersold R., Nogales E. Molecular architecture of human polycomb repressive complex 2. Elife. 2012; 1:e00005. 23110252 10.7554/eLife.00005 PMC3482686

[12] Holoch D., Margueron R. Mechanisms regulating PRC2 recruitment and enzymatic activity. Trends Biochem. Sci. 2017; 42:531–542. 28483375 10.1016/j.tibs.2017.04.003

[13] Chammas P., Mocavini I., Croce L.Di Engaging chromatin: PRC2 structure meets function. Br. J. Cancer. 2019; 122:315–328. 31708574 10.1038/s41416-019-0615-2 PMC7000746

[14] Laugesen A., Højfeldt J.W., Helin K. Molecular mechanisms directing PRC2 Recruitment and H3K27 Methylation. Mol. Cell. 2019; 74:8–18. 30951652 10.1016/j.molcel.2019.03.011 PMC6452890

[15] Bauer M., Trupke J., Ringrose L. The quest for mammalian Polycomb response elements: are we there yet?. Chromosoma. 2015; 125:471–496. 26453572 10.1007/s00412-015-0539-4 PMC4901126

[16] Tanay A., O’Donnell A.H., Damelin M., Bestor T.H. Hyperconserved CpG domains underlie Polycomb-binding sites. Proc. Natl. Acad. Sci. 2007; 104:5521–5526. 17376869 10.1073/pnas.0609746104 PMC1838490

[17] Kahn T.G., Dorafshan E., Schultheis D., Zare A., Stenberg P., Reim I., Pirrotta V., Schwartz Y.B. Interdependence of PRC1 and PRC2 for recruitment to Polycomb Response Elements. Nucleic Acids Res. 2016; 44:10132–10149. 27557709 10.1093/nar/gkw701 PMC5137424

[18] Fursova N.A., Blackledge N.P., Nakayama M., Ito S., Koseki Y., Farcas A.M., King H.W., Koseki H., Klose R.J. Synergy between Variant PRC1 complexes defines polycomb-mediated gene repression. Mol. Cell. 2019; 74:1020–1036. 31029541 10.1016/j.molcel.2019.03.024 PMC6561741

[19] Blackledge N.P., Fursova N.A., Kelley J.R., Huseyin M.K., Feldmann A., Klose R.J. PRC1 catalytic activity is central to polycomb system function. Mol. Cell. 2020; 77:857–874. 31883950 10.1016/j.molcel.2019.12.001 PMC7033600

[20] Tamburri S., Lavarone E., Fernández-Pérez D., Conway E., Zanotti M., Manganaro D., Pasini D. Histone H2AK119 mono-ubiquitination is essential for polycomb-mediated transcriptional repression. Mol. Cell. 2020; 77:840–856. 31883952 10.1016/j.molcel.2019.11.021 PMC7033561

[21] Zepeda-Martinez J.A., Pribitzer C., Wang J., Bsteh D., Golumbeanu S., Zhao Q., Burkard T.R., Reichholf B., Rhie S.K., Jude J. et al. Parallel PRC2/cPRC1 and vPRC1 pathways silence lineage-specific genes and maintain self-renewal in mouse embryonic stem cells. Sci. Adv. 2020; 6:eaax5692. 32270030 10.1126/sciadv.aax5692 PMC7112768

[22] Kasinath V., Beck C., Sauer P., Poepsel S., Kosmatka J., Faini M., Toso D., Aebersold R., Nogales E. JARID2 and AEBP2 regulate PRC2 in the presence of H2AK119ub1 and other histone modifications. Science. 2021; 371:eabc3393. 33479123 10.1126/science.abc3393 PMC7993630

[23] Højfeldt J.W., Hedehus L., Laugesen A., Tatar T., Wiehle L., Helin K. Non-core Subunits of the PRC2 complex are collectively required for its target-site specificity. Mol. Cell. 2019; 76:423–436. 31521506 10.1016/j.molcel.2019.07.031 PMC6842426

[24] Li H., Liefke R., Jiang J., Kurland J.V., Tian W., Deng P., Zhang W., He Q., Patel D.J., Bulyk M.L. et al. Polycomb-like proteins link the PRC2 complex to CpG islands. Nature. 2017; 549:287–291. 28869966 10.1038/nature23881 PMC5937281

[25] Healy E., Mucha M., Glancy E., Fitzpatrick D.J., Conway E., Neikes H.K., Monger C., Mierlo G.V, Baltissen M.P., Koseki Y. et al. PRC2.1 and PRC2.2 Synergize to Coordinate H3K27 Trimethylation. Mol. Cell. 2019; 76:437–452. 31521505 10.1016/j.molcel.2019.08.012

[26] Scelfo A., Fernández-Pérez D., Tamburri S., Zanotti M., Lavarone E., Soldi M., Bonaldi T., Ferrari K.J., Pasini D. Functional landscape of PCGF proteins reveals both RING1A/B-dependent-and RING1A/B-independent-specific activities. Mol. Cell. 2019; 74:1037–1052. 31029542 10.1016/j.molcel.2019.04.002 PMC6561742

[27] Endoh M., Endo T.A., Endoh T., Isono K., Sharif J., Ohara O., Toyoda T., Ito T., Eskeland R., Bickmore W.A. et al. Histone H2A mono-ubiquitination is a crucial step to mediate PRC1-dependent repression of developmental genes to maintain cell identity. PLOS Genet. 2012; 8:e1002774. 22844243 10.1371/journal.pgen.1002774 PMC3405999

[28] Gao Z., Lee P., Stafford J.M., Schimmelmann M., Schaefer A., Reinberg D. An AUTS2–Polycomb complex activates gene expression in the CNS. Nature. 2014; 516:349–354. 25519132 10.1038/nature13921 PMC4323097

[29] Zhao W., Huang Y., Zhang J., Liu M., Ji H., Wang C., Cao N., Li C., Xia Y., Jiang Q. et al. Polycomb group RING finger proteins 3/5 activate transcription via an interaction with the pluripotency factor Tex10 in embryonic stem cells. J. Biol. Chem. 2017; 292:21527–21537. 29054931 10.1074/jbc.M117.804054 PMC5766968

[30] Wang Q., Geng Z., Gong Y., Warren K., Zheng H., Imamura Y., Gao Z. WDR68 is essential for the transcriptional activation of the PRC1-AUTS2 complex and neuronal differentiation of mouse embryonic stem cells. Stem Cell Res. 2018; 33:206–214. 30448639 10.1016/j.scr.2018.10.023

[31] Frangini A., Sjöberg M., Roman-Trufero M., Dharmalingam G., Haberle V., Bartke T., Lenhard B., Malumbres M., Vidal M., Dillon N. The Aurora B Kinase and the Polycomb Protein Ring1B Combine to Regulate Active Promoters in Quiescent Lymphocytes. Mol. Cell. 2013; 51:647–661. 24034696 10.1016/j.molcel.2013.08.022

[32] Maezawa S., Hasegawa K., Yukawa M., Sakashita A., Alavattam K.G., Andreassen P.R., Vidal M., Koseki H., Barski A., Namekawa S.H. Polycomb directs timely activation of germline genes in spermatogenesis. Genes Dev. 2017; 31:1693–1703. 28924034 10.1101/gad.302000.117 PMC5647939

[33] Loubiere V., Papadopoulos G.L., Szabo Q., Martinez A.-M., Cavalli G. Widespread activation of developmental gene expression characterized by PRC1-dependent chromatin looping. Sci. Adv. 2020; 6:eaax4001. 31950077 10.1126/sciadv.aax4001 PMC6954061

[34] Leicher R., Ge E.J., Lin X., Reynolds M.J., Xie W., Walz T., Zhang B., Muir T.W., Liu S. Single-molecule and in silico dissection of the interaction between Polycomb repressive complex 2 and chromatin. Proc. Natl. Acad. Sci. 2020; 117:30465–30475. 33208532 10.1073/pnas.2003395117 PMC7720148

[35] Poepsel S., Kasinath V., Nogales E. Cryo-EM structures of PRC2 simultaneously engaged with two functionally distinct nucleosomes. Nat. Struct. Mol. Biol. 2018; 25:154–162. 29379173 10.1038/s41594-018-0023-y PMC5805599

[36] Grau D.J., Chapman B.A., Garlick J.D., Borowsky M., Francis N.J., Kingston R.E. Compaction of chromatin by diverse polycomb group proteins requires localized regions of high charge. Genes Dev. 2011; 25:2210–2221. 22012622 10.1101/gad.17288211 PMC3205590

[37] Matheson L., Elderkin S. Polycomb Bodies. Nuclear Architecture and Dynamics. 2018; Elsevier 297–320.

[38] Hernández-Muñoz I., Taghavi P., Kuijl C., Neefjes J., van Lohuizen M. Association of BMI1 with Polycomb Bodies Is Dynamic and Requires PRC2/EZH2 and the Maintenance DNA Methyltransferase DNMT1. Mol. Cell. Biol. 2005; 25:11047–11058. 16314526 10.1128/MCB.25.24.11047-11058.2005 PMC1316945

[39] Messmer S., Franke A., Paro R. Analysis of the functional role of the Polycomb chromo domain in Drosophila melanogaster. Genes Dev. 1992; 6:1241–1254. 1628830 10.1101/gad.6.7.1241

[40] Ren X., Vincenz C., Kerppola T.K. Changes in the Distributions and Dynamics of Polycomb Repressive Complexes during Embryonic Stem Cell Differentiation. Mol. Cell. Biol. 2008; 28:2884–2895. 18316406 10.1128/MCB.00949-07 PMC2293085

[41] Isono K., Endo T.A., Ku M., Yamada D., Suzuki R., Sharif J., Ishikura T., Toyoda T., Bernstein B.E., Koseki H. SAM domain polymerization links subnuclear clustering of PRC1 to gene silencing. Dev. Cell. 2013; 26:565–577. 24091011 10.1016/j.devcel.2013.08.016

[42] Saurin A.J., Shiels C., Williamson J., Satijn D.P.E., Otte A.P., Sheer D., Freemont P.S. The human polycomb group complex associates with pericentromeric heterochromatin to form a novel nuclear domain. J. Cell Biol. 1998; 142:887–898. 9722603 10.1083/jcb.142.4.887 PMC2132874

[43] Buchenau P., Hodgson J., Strutt H., Arndt-Jovin D.J. The distribution of polycomb-group proteins during cell division and development in Drosophila embryos: Impact on models for silencing. J. Cell Biol. 1998; 141:469–481. 9548724 10.1083/jcb.141.2.469 PMC2148446

[44] Moussa H.F., Bsteh D., Yelagandula R., Pribitzer C., Stecher K., Bartalska K., Michetti L., Wang J., Zepeda-Martinez J.A., Elling U. et al. Canonical PRC1 controls sequence-independent propagation of Polycomb-mediated gene silencing. Nat. Commun. 2019; 10:1–12. 31036804 10.1038/s41467-019-09628-6 PMC6488670

[45] Kloet S.L., Makowski M.M., Baymaz H.I., Van Voorthuijsen L., Karemaker I.D., Santanach A., Jansen P.W.T.C., Di Croce L., Vermeulen M. The dynamic interactome and genomic targets of Polycomb complexes during stem-cell differentiation. Nat. Struct. Mol. Biol. 2016; 23:682–690. 27294783 10.1038/nsmb.3248 PMC4939079

[46] Endoh M., Endo T.A., Endoh T., Fujimura Y.I., Ohara O., Toyoda T., Otte A.P., Okano M., Brockdorff N., Vidal M. et al. Polycomb group proteins Ring1A/B are functionally linked to the core transcriptional regulatory circuitry to maintain ES cell identity. Development. 2008; 135:1513–1524. 18339675 10.1242/dev.014340

[47] Cao R., Tsukada Y.I., Zhang Y. Role of Bmi-1 and Ring1A in H2A Ubiquitylation and Hox Gene Silencing. Mol. Cell. 2005; 20:845–854. 16359901 10.1016/j.molcel.2005.12.002

[48] Li Z., Cao R., Wang M., Myers M.P., Zhang Y., Xu R.-M. Structure of a Bmi-1-Ring1B Polycomb Group Ubiquitin Ligase Complex *. J. Biol. Chem. 2006; 281:20643–20649. 16714294 10.1074/jbc.M602461200

[49] Buchwald G., Stoop P., Weichenrieder O., Perrakis A., Lohuizen M., Sixma T.K. Structure and E3-ligase activity of the Ring–Ring complex of Polycomb proteins Bmi1 and Ring1b. EMBO J. 2006; 25:2465–2474. 16710298 10.1038/sj.emboj.7601144 PMC1478191

[50] McGinty R.K., Henrici R.C., Tan S. Crystal structure of the PRC1 ubiquitylation module bound to the nucleosome. Nature. 2014; 514:591–596. 25355358 10.1038/nature13890 PMC4215650

[51] Bezsonova I., Walker J.R., Bacik J.P., Duan S., Dhe-Paganon S., Arrowsmith C.H. Ring1B Contains a Ubiquitin-Like Docking Module for Interaction with Cbx Proteins. Biochemistry. 2009; 48:10542–10548. 19791798 10.1021/bi901131u

[52] Chittock E.C., Latwiel S., Miller T.C.R., Müller C.W. Molecular architecture of polycomb repressive complexes. Biochem. Soc. Trans. 2017; 45:193–205. 28202673 10.1042/BST20160173 PMC5310723

[53] Wang R., Taylor A.B., Leal B.Z., Chadwell L.V., Ilangovan U., Robinson A.K., Schirf V., Hart P.J., Lafer E.M., Demeler B. et al. Polycomb group targeting through different binding partners of RING1B C-terminal domain. Structure. 2010; 18:966–975. 20696397 10.1016/j.str.2010.04.013 PMC4445678

[54] Kaustov L., Ouyang H., Amaya M., Lemak A., Nady N., Duan S., Wasney G.A., Li Z., Vedadi M., Schapira M. et al. Recognition and Specificity Determinants of the Human Cbx Chromodomains *. J. Biol. Chem. 2011; 286:521–529. 21047797 10.1074/jbc.M110.191411 PMC3013012

[55] Völkel P., le Faou P., Vandamme J., Pira D., Angrand P.O. A human polycomb isoform lacking the Pc box does not participate to PRC1 complexes BUT forms protein assemblies and represses transcription. Epigenetics. 2012; 7:482–491. 22419124 10.4161/epi.19741

[56] Bernstein E., Duncan E.M., Masui O., Gil J., Heard E., Allis C.D. Mouse Polycomb Proteins Bind Differentially to Methylated Histone H3 and RNA and Are Enriched in Facultative Heterochromatin. Mol. Cell. Biol. 2006; 26:2560–2569. 16537902 10.1128/MCB.26.7.2560-2569.2006 PMC1430336

[57] Connelly K.E., Weaver T.M., Alpsoy A., Gu B.X., Musselman C.A., Dykhuizen E.C. Engagement of DNA and H3K27me3 by the CBX8 chromodomain drives chromatin association. Nucleic Acids Res. 2019; 47:2289–2305. 30597065 10.1093/nar/gky1290 PMC6411926

[58] Plys A.J., Davis C.P., Kim J., Rizki G., Keenen M.M., Marr S.K., Kingston R.E. Phase separation of polycomb-repressive complex 1 is governed by a charged disordered region of CBX2. Genes Dev. 2019; 33:799–813. 31171700 10.1101/gad.326488.119 PMC6601514

[59] Kagey M.H., Melhuish T.A., Wotton D. The polycomb protein Pc2 is a SUMO E3. Cell. 2003; 113:127–137. 12679040 10.1016/s0092-8674(03)00159-4

[60] Li B., Zhou J., Liu P., Hu J., Jin H., Shimono Y., Takahashi M., Xu G. Polycomb protein Cbx4 promotes SUMO modification of de novo DNA methyltransferase Dnmt3a. Biochem. J. 2007; 405:369–378. 17439403 10.1042/BJ20061873 PMC1904525

[61] Ismail I.H., Gagné J.P., Caron M.C., McDonald D., Xu Z., Masson J.Y., Poirier G.G., Hendzel M.J. CBX4-mediated SUMO modification regulates BMI1 recruitment at sites of DNA damage. Nucleic Acids Res. 2012; 40:5497–5510. 22402492 10.1093/nar/gks222 PMC3384338

[62] Hemenway C.S., De Erkenez A.C., Gould G.C.D. The polycomb protein MPc3 interacts with AF9, an MLL fusion partner in t(9;11)(p22;q23) acute leukemias. Oncogene. 2001; 20:3798–3805. 11439343 10.1038/sj.onc.1204478

[63] Connelly K.E., Dykhuizen E.C. Compositional and functional diversity of canonical PRC1 complexes in mammals. Biochim. Biophys. Acta - Gene Regul. Mech. 2017; 1860:233–245. 28007606 10.1016/j.bbagrm.2016.12.006

[64] Kawaguchi T., Machida S., Kurumizaka H., Tagami H., Nakayama J. Phosphorylation of CBX2 controls its nucleosome-binding specificity. J. Biochem. 2017; 162:343–355. 28992316 10.1093/jb/mvx040

[65] Maethner E., Garcia-Cuellar M.P., Breitinger C., Takacova S., Divoky V., Hess J.L., Slany R.K. MLL-ENL Inhibits Polycomb Repressive Complex 1 to Achieve Efficient Transformation of Hematopoietic Cells. Cell Rep. 2013; 3:1553–1566. 23623499 10.1016/j.celrep.2013.03.038 PMC4124133

[66] Malik B., Hemenway C.S. CBX8, a component of the Polycomb PRC1 complex, modulates DOT1L-mediated gene expression through AF9/MLLT3. FEBS Lett. 2013; 587:3038–3044. 23891621 10.1016/j.febslet.2013.07.034 PMC3800029

[67] Tan J., Jones M., Koseki H., Nakayama M., Muntean A.G., Maillard I., Hess J.L. CBX8, a Polycomb Group Protein, Is Essential for MLL-AF9-Induced Leukemogenesis. Cancer Cell. 2011; 20:563–575. 22094252 10.1016/j.ccr.2011.09.008 PMC3220883

[68] Seif E., Kang J.J., Sasseville C., Senkovich O., Kaltashov A., Boulier E.L., Kapur I., Kim C.A., Francis N.J. Phase separation by the polyhomeotic sterile alpha motif compartmentalizes Polycomb Group proteins and enhances their activity. Nat. Commun. 2020; 11:1–19. 33154383 10.1038/s41467-020-19435-z PMC7644731

[69] Wani A.H., Boettiger A.N., Schorderet P., Ergun A., Münger C., Sadreyev R.I., Zhuang X., Kingston R.E., Francis N.J. Chromatin topology is coupled to Polycomb group protein subnuclear organization. Nat. Commun. 2016; 7:1–13. 10.1038/ncomms10291 PMC4735512 26759081

[70] Robinson A.K., Leal B.Z., Chadwell L.V., Wang R., Ilangovan U., Kaur Y., Junco S.E., Schirf V., Osmulski P.A., Gaczynska M. et al. The growth-suppressive function of the polycomb group protein polyhomeotic is mediated by polymerization of its sterile alpha motif (SAM) domain. J. Biol. Chem. 2012; 287:8702–8713. 22275371 10.1074/jbc.M111.336115 PMC3308824

[71] Kim C.A., Francis N.J. Chromatin: Polycomb Group SAMs Unite. Curr. Biol. 2016; 26:R710–R712. 27505242 10.1016/j.cub.2016.06.001

[72] Saurin A.J., Shao Z., Erdjument-Bromage H., Tempst P., Kingston R.E. A drosophila polycomb group complex includes zeste and dTAFII proteins. Nature. 2001; 412:655–660. 11493925 10.1038/35088096

[73] Levine S.S., Weiss A., Erdjument-Bromage H., Shao Z., Tempst P., Kingston R.E. The Core of the Polycomb Repressive Complex Is Compositionally and Functionally Conserved in Flies and Humans. Mol. Cell. Biol. 2002; 22:6070–6078. 12167701 10.1128/MCB.22.17.6070-6078.2002 PMC134016

[74] Li M., Zhang S., Zhao W., Hou C., Ma X., Li X., Huang B., Chen H., Chen D. RYBP modulates stability and function of Ring1B through targeting UBE3A. FASEB J. 2019; 33:683–695. 30040487 10.1096/fj.201800397R

[75] Rose N.R., King H.W., Blackledge N.P., Fursova N.A., Ember K.J., Fischer R., Kessler B.M., Klose R.J. RYBP stimulates PRC1 to shape chromatin-based communication between polycomb repressive complexes. Elife. 2016; 5:e18591. 27705745 10.7554/eLife.18591 PMC5065315

[76] Zhao J., Wang M., Chang L., Yu J., Song A., Liu C., Huang W., Zhang T., Wu X., Shen X. et al. RYBP/YAF2-PRC1 complexes and histone H1-dependent chromatin compaction mediate propagation of H2AK119ub1 during cell division. Nat. Cell Biol. 2020; 22:439–452. 32203418 10.1038/s41556-020-0484-1

[77] Blackledge N.P., Farcas A.M., Kondo T., King H.W., McGouran J.F., Hanssen L.L.P., Ito S., Cooper S., Kondo K., Koseki Y. et al. Variant PRC1 Complex-Dependent H2A Ubiquitylation Drives PRC2 recruitment and polycomb domain formation. Cell. 2014; 157:1445–1459. 24856970 10.1016/j.cell.2014.05.004 PMC4048464

[78] Frescas D., Guardavaccaro D., Bassermann F., Koyama-Nasu R., Pagano M. JHDM1B/FBXL10 is a nucleolar protein that represses transcription of ribosomal RNA genes. Nature. 2007; 450:309–313. 17994099 10.1038/nature06255

[79] Tsukada Y.I., Fang J., Erdjument-Bromage H., Warren M.E., Borchers C.H., Tempst P., Zhang Y. Histone demethylation by a family of JmjC domain-containing proteins. Nature. 2006; 439:811–816. 16362057 10.1038/nature04433

[80] Wang Z., Gearhart M.D., Lee Y.W., Kumar I., Ramazanov B., Zhang Y., Hernandez C., Lu A.Y., Neuenkirchen N., Deng J. et al. A Non-canonical BCOR-PRC1.1 Complex Represses Differentiation Programs in Human ESCs. Cell Stem Cell. 2018; 22:235–251. 29337181 10.1016/j.stem.2017.12.002 PMC5797497

[81] Wong S.J., Senkovich O., Artigas J.A., Gearhart M.D., Ilangovan U., Graham D.W., Abel K.N., Yu T., Hinck A.P., Bardwell V.J. et al. Structure and Role of BCOR PUFD in Noncanonical PRC1 Assembly and Disease. Biochemistry. 2020; 59:2718–2728. 32628469 10.1021/acs.biochem.0c00285 PMC8544800

[82] Cascoń A., Robledo M. MAX and MYC: A heritable breakup. Cancer Res. 2012; 72:3119–3124. 22706201 10.1158/0008-5472.CAN-11-3891

[83] Hurlin P.J., Steingrìmsson E., Copeland N.G., Jenkins N.A., Eisenman R.N. Mga, a dual-specificity transcription factor that interacts with Max and contains a T-domain DNA-binding motif. EMBO J. 2000; 19:3841–3842. 10.1093/emboj/18.24.7019 PMC1171765 10601024

[84] Stielow B., Finkernagel F., Stiewe T., Nist A., Suske G. MGA, L3MBTL2 and E2F6 determine genomic binding of the non-canonical Polycomb repressive complex PRC1.6. PLoS Genet. 2018; 14:e1007193. 29381691 10.1371/journal.pgen.1007193 PMC5806899

[85] Trojer P., Cao A.R., Gao Z., Li Y., Zhang J., Xu X., Li G., Losson R., Erdjument-Bromage H., Tempst P. et al. L3MBTL2 Protein Acts in Concert with PcG Protein-Mediated Monoubiquitination of H2A to Establish a Repressive Chromatin Structure. Mol. Cell. 2011; 42:438–450. 21596310 10.1016/j.molcel.2011.04.004 PMC3142354

[86] Kuzmichev A., Nishioka K., Erdjument-Bromage H., Tempst P., Reinberg D. Histone methyltransferase activity associated with a human multiprotein complex containing the enhancer of zeste protein. Genes Dev. 2002; 16:2893–2905. 12435631 10.1101/gad.1035902 PMC187479

[87] Morin R.D., Johnson N.A., Severson T.M., Mungall A.J., An J., Goya R., Paul J.E., Boyle M., Woolcock B.W., Kuchenbauer F. et al. Somatic mutations altering EZH2 (Tyr641) in follicular and diffuse large B-cell lymphomas of germinal-center origin. Nat. Genet. 2010; 42:181–185. 20081860 10.1038/ng.518 PMC2850970

[88] Sneeringer C.J., Scott M.P., Kuntz K.W., Knutson S.K., Pollock R.M., Richon V.M., Copeland R.A. Coordinated activities of wild-type plus mutant EZH2 drive tumor-associated hypertrimethylation of lysine 27 on histone H3 (H3K27) in human B-cell lymphomas. Proc. Natl. Acad. Sci. 2010; 107:20980–20985. 21078963 10.1073/pnas.1012525107 PMC3000297

[89] McCabe M.T., Graves A.P., Ganji G., Diaz E., Halsey W.S., Jiang Y., Smitheman K.N., Ott H.M., Pappalardi M.B., Allen K.E. et al. Mutation of A677 in histone methyltransferase EZH2 in human B-cell lymphoma promotes hypertrimethylation of histone H3 on lysine 27 (H3K27). Proc. Natl. Acad. Sci. 2012; 109:2989–2994. 22323599 10.1073/pnas.1116418109 PMC3287005

[90] Majer C.R., Jin L., Scott M.P., Knutson S.K., Kuntz K.W., Keilhack H., Smith J.J., Moyer M.P., Richon V.M., Copeland R.A. et al. A687V EZH2 is a gain-of-function mutation found in lymphoma patients. FEBS Lett. 2012; 586:3448–3451. 22850114 10.1016/j.febslet.2012.07.066

[91] Cao R., Zhang Y. SUZ12 is required for both the histone methyltransferase activity and the silencing function of the EED-EZH2 complex. Mol. Cell. 2004; 15:57–67. 15225548 10.1016/j.molcel.2004.06.020

[92] Zhang Q., Vo N., Goodman R.H. Histone Binding Protein RbAp48 Interacts with a Complex of CREB Binding Protein and Phosphorylated CREB. Mol. Cell. Biol. 2000; 20:4970–4978. 10866654 10.1128/mcb.20.14.4970-4978.2000 PMC85947

[93] Sun J.M., Hou Y.C., Davie J.R. Differential distribution of unmodified and phosphorylated histone deacetylase 2 in chromatin. J. Biol. Chem. 2007; 282:33227–33236. 17827154 10.1074/jbc.M703549200

[94] Mahajan M.C., Narlikar G.J., Boyapaty G., Kingston R.E., Weissman S.M. Heterogeneous nuclear ribonucleoprotein C1/C2, MeCP1, and SWI/SNF form a chromatin remodeling complex at the β-globin locus control region. Proc. Natl. Acad. Sci. 2005; 102:15012–15017. 16217013 10.1073/pnas.0507596102 PMC1257739

[95] Ballaré C., Lange M., Lapinaite A., Martin G.M., Morey L., Pascual G., Liefke R., Simon B., Shi Y., Gozani O. et al. Phf19 links methylated Lys36 of histone H3 to regulation of Polycomb activity. Nat. Struct. Mol. Biol. 2012; 19:1257–1265. 23104054 10.1038/nsmb.2434 PMC3926938

[96] Boulay G., Rosnoblet C., Guérardel C., Angrand P.O., Leprince D. Functional characterization of human Polycomb-like 3 isoforms identifies them as components of distinct EZH2 protein complexes. Biochem. J. 2011; 434:333–342. 21143197 10.1042/BJ20100944

[97] Kalb R., Latwiel S., Baymaz H.I., Jansen P.W.T.C., Müller C.W., Vermeulen M., Müller J. Histone H2A monoubiquitination promotes histone H3 methylation in Polycomb repression. Nat. Struct. Mol. Biol. 2014; 21:569–571. 24837194 10.1038/nsmb.2833

[98] Kim H., Kang K., Kim J. AEBP2 as a potential targeting protein for Polycomb Repression Complex PRC2. Nucleic Acids Res. 2009; 37:2940–2950. 19293275 10.1093/nar/gkp149 PMC2685092

[99] Grijzenhout A., Godwin J., Koseki H., Gdula M.R., Szumska D., McGouran J.F., Bhattacharya S., Kessler B.M., Brockdorff N., Cooper S. Functional analysis of AEBP2, a PRC2 Polycomb protein, reveals a Trithorax phenotype in embryonic development and in ESCs. Development. 2016; 143:2716–2723. 27317809 10.1242/dev.123935 PMC5004903

[100] He G.P., Kim S., Ro H.S. Cloning and characterization of a novel zinc finger transcriptional repressor. A direct role of the zinc finger motif in repression. J. Biol. Chem. 1999; 274:14678–14684. 10329662 10.1074/jbc.274.21.14678

[101] Li G., Margueron R., Ku M., Chambon P., Bernstein B.E., Reinberg D. Jarid2 and PRC2, partners in regulating gene expression. Genes Dev. 2010; 24:368–380. 20123894 10.1101/gad.1886410 PMC2816736

[102] Sanulli S., Justin N., Teissandier A., Ancelin K., Portoso M., Caron M., Michaud A., Lombard B., da Rocha S.T., Offer J. et al. Jarid2 Methylation via the PRC2 Complex Regulates H3K27me3 Deposition during Cell Differentiation. Mol. Cell. 2015; 57:769–783. 25620564 10.1016/j.molcel.2014.12.020 PMC4352895

[103] Conway E., Jerman E., Healy E., Ito S., Holoch D., Oliviero G., Deevy O., Glancy E., Fitzpatrick D.J., Mucha M. et al. A Family of Vertebrate-Specific Polycombs Encoded by the LCOR/LCORL Genes Balance PRC2 Subtype Activities. Mol. Cell. 2018; 70:408–421. 29628311 10.1016/j.molcel.2018.03.005

[104] Zhang Q., Agius S.C., Flanigan S.F., Uckelmann M., Levina V., Owen B.M., Davidovich C. PALI1 facilitates DNA and nucleosome binding by PRC2 and triggers an allosteric activation of catalysis. Nat. Commun. 2021; 12:1–18. 34321472 10.1038/s41467-021-24866-3 PMC8319299

[105] Malte Beringer A., Pisano P., Di Carlo V., Payer B., Wierer M., Di Croce Correspondence L. EPOP functionally links elongin and polycomb in pluripotent stem cells. Mol. Cell. 2016; 64:645–658. 27863225 10.1016/j.molcel.2016.10.018

[106] Piunti A., Smith E.R., Morgan M.A.J., Ugarenko M., Khaltyan N., Helmin K.A., Ryan C.A., Murray D.C., Rickels R.A., Yilmaz B.D. et al. CATACOMB: An endogenous inducible gene that antagonizes H3K27 methylation activity of Polycomb repressive complex 2 via an H3K27M-like mechanism. Sci. Adv. 2019; 5:eaax2887. 31281901 10.1126/sciadv.aax2887 PMC6609211

[107] Ragazzini R., Pérez-Palacios R., Baymaz I.H., Diop S., Ancelin K., Zielinski D., Michaud A., Givelet M., Borsos M., Aflaki S. et al. EZHIP constrains Polycomb Repressive Complex 2 activity in germ cells. Nat. Commun. 2019; 10:1–18. 31451685 10.1038/s41467-019-11800-x PMC6710278

[108] Jain S.U., Rashoff A.Q., Krabbenhoft S.D., Hoelper D., Do T.J., Gibson T.J., Lundgren S.M., Bondra E.R., Deshmukh S., Harutyunyan A.S. et al. H3 K27M and EZHIP Impede H3K27-Methylation spreading by inhibiting allosterically stimulated PRC2. Mol. Cell. 2020; 80:726–735. 33049227 10.1016/j.molcel.2020.09.028 PMC7680438

[109] Baylin S.B., Jones P.A. Epigenetic determinants of cancer. Cold Spring Harb. Perspect. Biol. 2016; 8:a019505. 27194046 10.1101/cshperspect.a019505 PMC5008069

[110] Lee T.I., Jenner R.G., Boyer L.A., Guenther M.G., Levine S.S., Kumar R.M., Chevalier B., Johnstone S.E., Cole M.F., Isono K. et al. Control of developmental regulators by polycomb in human embryonic stem cells. Cell. 2006; 125:301–313. 16630818 10.1016/j.cell.2006.02.043 PMC3773330

[111] Kamminga L.M., Bystrykh L.V., De Boer A., Houwer S., Douma J., Weersing E., Dontje B., De Haan G. The Polycomb group gene Ezh2 prevents hematopoietic stem cell exhaustion. Blood. 2006; 107:2170–2179. 16293602 10.1182/blood-2005-09-3585 PMC1895717

[112] Wen Y., Cai J., Hou Y., Huang Z., Wang Z. Role of EZH2 in cancer stem cells: From biological insight to a therapeutic target. Oncotarget. 2017; 8:37974–37990. 28415635 10.18632/oncotarget.16467 PMC5514966

[113] Popov N., Gil J. Epigenetic regulation of the INK4B-ARF-INK4a locus: In sickness and in health. Epigenetics. 2010; 5:685–690. 20716961 10.4161/epi.5.8.12996 PMC3052884

[114] Bracken A.P., Helin K. Polycomb group proteins: Navigators of lineage pathways led astray in cancer. Nat. Rev. Cancer. 2009; 9:773–784. 19851313 10.1038/nrc2736

[115] Wang M.C., Li C.L., Cui J., Jiao M., Wu T., Jing L., Nan K.J. BMI-1, a promising therapeutic target for human cancer (Review). Oncol. Lett. 2015; 10:583–588. 26622537 10.3892/ol.2015.3361 PMC4509079

[116] Wu H.-X., Wang Z.-X., Zhao Q., Chen D.-L., He M.-M., Yang L.-P., Wang Y.-N., Jin Y., Ren C., Luo H.-Y. et al. Tumor mutational and indel burden: a systematic pan-cancer evaluation as prognostic biomarkers. Ann. Transl. Med. 2019; 7:640–640. 31930041 10.21037/atm.2019.10.116 PMC6944566

[117] Llabata P., Mitsuishi Y., Choi P.S., Cai D., Francis J.M., Torres-Diz M., Udeshi N.D., Golomb L., Wu Z., Zhou J. et al. Multi-Omics Analysis Identifies MGA as a Negative Regulator of the MYC Pathway in Lung Adenocarcinoma. Mol. Cancer Res. 2020; 18:574–584. 31862696 10.1158/1541-7786.MCR-19-0657 PMC7219472

[118] Mathsyaraja H., Catchpole J., Freie B., Eastwood E., Babaeva E., Geuenich M., Cheng P.F., Ayers J., Yu M., Wu N. et al. Loss of MGA repression mediated by an atypical polycomb complex promotes tumor progression and invasiveness. Elife. 2021; 10:e64212. 34236315 10.7554/eLife.64212 PMC8266391

[119] Astolfi A., Fiore M., Melchionda F., Indio V., Bertuccio S.N., Pession A. BCOR involvement in cancer. Epigenomics. 2019; 11:835–855. 31150281 10.2217/epi-2018-0195 PMC6595546

[120] Wamstad J.A., Bardwell V.J. Characterization of Bcor expression in mouse development. Gene Expr. Patterns. 2007; 7:550–557. 17344103 10.1016/j.modgep.2007.01.006 PMC2002546

[121] Hoffman L.M., DeWire M., Ryall S., Buczkowicz P., Leach J., Miles L., Ramani A., Brudno M., Kumar S.S., Drissi R. et al. Spatial genomic heterogeneity in diffuse intrinsic pontine and midline high-grade glioma: implications for diagnostic biopsy and targeted therapeutics. Acta Neuropathol. Commun. 2016; 4:1–8. 26727948 10.1186/s40478-015-0269-0 PMC4700584

[122] Argani P., Kao Y.C., Zhang L., Bacchi C., Matoso A., Alaggio R., Epstein J.I., Antonescu C.R. Primary Renal Sarcomas with BCOR-CCNB3 Gene Fusion. Am. J. Surg. Pathol. 2017; 41:1702–1712. 28817404 10.1097/PAS.0000000000000926 PMC5680139

[123] McEvoy J., Nagahawatte P., Finkelstein D., Richards-Yutz J., Valentine M., Ma J., Mullighan C., Song G., Chen X., Wilson M. et al. RB1 gene inactivation by chromothripsis in human retinoblastoma. Oncotarget. 2014; 5:438–450. 24509483 10.18632/oncotarget.1686 PMC3964219

[124] Grossmann V., Tiacci E., Holmes A.B., Kohlmann A., Martelli M.P., Kern W., Spanhol-Rosseto A., Klein H.U., Dugas M., Schindela S. et al. Whole-exome sequencing identifies somatic mutations of BCOR in acute myeloid leukemia with normal karyotype. Blood. 2011; 118:6153–6163. 22012066 10.1182/blood-2011-07-365320

[125] Lindsley R.C., Mar B.G., Mazzola E., Grauman P.V., Shareef S., Allen S.L., Pigneux A., Wetzler M., Stuart R.K., Erba H.P. et al. Acute myeloid leukemia ontogeny is defined by distinct somatic mutations. Blood. 2015; 125:1367–1376. 25550361 10.1182/blood-2014-11-610543 PMC4342352

[126] Damm F., Chesnais V., Nagata Y., Yoshida K., Scourzic L., Okuno Y., Itzykson R., Sanada M., Shiraishi Y., Gelsi-Boyer V. et al. BCOR and BCORL1 mutations in myelodysplastic syndromes and related disorders. Blood. 2013; 122:3169–3177. 24047651 10.1182/blood-2012-11-469619

[127] Dobashi A., Tsuyama N., Asaka R., Togashi Y., Ueda K., Sakata S., Baba S., Sakamoto K., Hatake K., Takeuchi K. Frequent BCOR aberrations in extranodal NK/T-Cell lymphoma, nasal type. Genes Chromosom. Cancer. 2016; 55:460–471. 26773734 10.1002/gcc.22348

[128] Isshiki Y., Iwama A. Emerging role of noncanonical polycomb repressive complexes in normal and malignant hematopoiesis. Exp. Hematol. 2018; 68:10–14. 30554637 10.1016/j.exphem.2018.10.008

[129] Cao Q., Gearhart M.D., Gery S., Shojaee S., Yang H., Sun H., Lin D.C., Bai J.W., Mead M., Zhao Z. et al. BCOR regulates myeloid cell proliferation and differentiation. Leukemia. 2016; 30:1155–1165. 26847029 10.1038/leu.2016.2 PMC5131645

[130] Kolla L., Kelly M.C., Mann Z.F., Anaya-Rocha A., Ellis K., Lemons A., Palermo A.T., So K.S., Mays J.C., Orvis J. et al. Characterization of the development of the mouse cochlear epithelium at the single cell level. Nat. Commun. 2020; 11:1–16. 32404924 10.1038/s41467-020-16113-y PMC7221106

[131] Iwama A. Polycomb repressive complexes in hematological malignancies. Blood. 2017; 130:23–29. 28483764 10.1182/blood-2017-02-739490

[132] Raedt T.De, Beert E., Pasmant E., Luscan A., Brems H., Ortonne N., Helin K., Hornick J.L., Mautner V., Kehrer-Sawatzki H. et al. PRC2 loss amplifies Ras-driven transcription and confers sensitivity to BRD4-based therapies. Nature. 2014; 514:247–251. 25119042 10.1038/nature13561

[133] Ernst T., Chase A.J., Score J., Hidalgo-Curtis C.E., Bryant C., Jones A.V, Waghorn K., Zoi K., Ross F.M., Reiter A. et al. Inactivating mutations of the histone methyltransferase gene EZH2 in myeloid disorders. Nat. Genet. 2010; 42:722–726. 20601953 10.1038/ng.621

[134] Nikoloski G., Langemeijer S.M.C., Kuiper R.P., Knops R., Massop M., Tönnissen E.R.L.T.M., Heijden A., Scheele T.N., Vandenberghe P., Witte T. et al. Somatic mutations of the histone methyltransferase gene EZH2 in myelodysplastic syndromes. Nat. Genet. 2010; 42:665–667. 20601954 10.1038/ng.620

[135] Bejar R., Stevenson K., Abdel-Wahab O., Galili N., Nilsson B., Garcia-Manero G., Kantarjian H., Raza A., Levine R.L., Neuberg D. et al. Clinical effect of point mutations in myelodysplastic syndromes. N. Engl. J. Med. 2011; 364:2496–2506. 21714648 10.1056/NEJMoa1013343 PMC3159042

[136] Mochizuki-Kashio M., Aoyama K., Sashida G., Oshima M., Tomioka T., Muto T., Wang C., Iwama A. Ezh2 loss in hematopoietic stem cells predisposes mice to develop heterogeneous malignancies in an Ezh1-dependent manner. Blood. 2015; 126:1172–1183. 26219303 10.1182/blood-2015-03-634428

[137] Shirahata-Adachi M., Iriyama C., Tomita A., Suzuki Y., Shimada K., Kiyoi H. Altered EZH2 splicing and expression is associated with impaired histone H3 lysine 27 tri-Methylation in myelodysplastic syndrome. Leuk. Res. 2017; 63:90–97. 29127861 10.1016/j.leukres.2017.10.015

[138] Gangat N., Mudireddy M., Lasho T.L., Finke C.M., Nicolosi M., Szuber N., Patnaik M.M., Pardanani A., Hanson C.A., Ketterling R.P. et al. Mutations and prognosis in myelodysplastic syndromes: karyotype-adjusted analysis of targeted sequencing in 300 consecutive cases and development of a genetic risk model. Am. J. Hematol. 2018; 93:691–697. 29417633 10.1002/ajh.25064

[139] Wang C., Mochel N.S.R., Christenson S.A., Cassandras M., Moon R., Brumwell A.N., Byrnes L.E., Li A., Yokosaki Y., Shan P. et al. Expansion of hedgehog disrupts mesenchymal identity and induces emphysema phenotype. J. Clin. Invest. 2018; 128:4343–4358. 29999500 10.1172/JCI99435 PMC6159975

[140] Korfhage J., Lombard D.B. Malignant Peripheral Nerve Sheath Tumors: From Epigenome to Bedside. Mol. Cancer Res. 2019; 17:1417–1428. 31023785 10.1158/1541-7786.MCR-19-0147 PMC6610818

[141] Marchione D.M., Lisby A., Viaene A.N., Santi M., Nasrallah M., Wang L.-P., Williams E.A., Larque A.B., Chebib I., Garcia B.A. et al. Histone H3K27 dimethyl loss is highly specific for malignant peripheral nerve sheath tumor and distinguishes true PRC2 loss from isolated H3K27 trimethyl loss. Mod. Pathol. 2019; 32:1434–1446. 31175328 10.1038/s41379-019-0287-8 PMC6763358

[142] Khuong-Quang D.-A., Buczkowicz P., Rakopoulos P., Liu X.-Y., Fontebasso A.M., Bouffet E., Bartels U., Albrecht S., Schwartzentruber J., Letourneau L. et al. K27M mutation in histone H3.3 defines clinically and biologically distinct subgroups of pediatric diffuse intrinsic pontine gliomas. Acta Neuropathol. 2012; 124:439–447. 22661320 10.1007/s00401-012-0998-0 PMC3422615

[143] Schwartzentruber J., Korshunov A., Liu X.-Y., Jones D.T.W., Pfaff E., Jacob K., Sturm D., Fontebasso A.M., Quang D.-A.K., Tönjes M. et al. Driver mutations in histone H3.3 and chromatin remodelling genes in paediatric glioblastoma. Nature. 2012; 482:226–231. 22286061 10.1038/nature10833

[144] Fontebasso A.M., Papillon-Cavanagh S., Schwartzentruber J., Nikbakht H., Gerges N., Fiset P.-O., Bechet D., Faury D., Jay N.De, Ramkissoon L.A. et al. Recurrent somatic mutations in ACVR1 in pediatric midline high-grade astrocytoma. Nat. Genet. 2014; 46:462–466. 24705250 10.1038/ng.2950 PMC4282994

[145] Taylor K.R., Mackay A., Truffaux N., Butterfield Y.S., Morozova O., Philippe C., Castel D., Grasso C.S., Vinci M., Carvalho D. et al. Recurrent activating ACVR1 mutations in diffuse intrinsic pontine glioma. Nat. Genet. 2014; 46:457–461. 24705252 10.1038/ng.2925 PMC4018681

[146] Bender S., Tang Y., Lindroth A.M., Hovestadt V., Jones D.T.W., Kool M., Zapatka M., Northcott P.A., Sturm D., Wang W. et al. Reduced H3K27me3 and DNA hypomethylation are major drivers of gene expression in K27M mutant pediatric high-grade gliomas. Cancer Cell. 2013; 24:660–672. 24183680 10.1016/j.ccr.2013.10.006

[147] Lewis P.W., Müller M.M., Koletsky M.S., Cordero F., Lin S., Banaszynski L.A., Garcia B.A., Muir T.W., Becher O.J., Allis C.D. Inhibition of PRC2 Activity by a Gain-of-Function H3 Mutation Found in Pediatric Glioblastoma. Science. 2013; 340:857–861. 23539183 10.1126/science.1232245 PMC3951439

[148] Venneti S., Garimella M.T., Sullivan L.M., Martinez D., Huse J.T., Heguy A., Santi M., Thompson C.B., Judkins A.R. Evaluation of Histone 3 Lysine 27 Trimethylation (H3K27me3) and Enhancer of Zest 2 (EZH2) in Pediatric Glial and Glioneuronal Tumors Shows Decreased H3K27me3 in H3F3A K27M Mutant Glioblastomas. Brain Pathol. 2013; 23:558–564. 23414300 10.1111/bpa.12042 PMC3701028

[149] Chan K.-M., Fang D., Gan H., Hashizume R., Yu C., Schroeder M., Gupta N., Mueller S., James C.D., Jenkins R. et al. The histone H3.3K27M mutation in pediatric glioma reprograms H3K27 methylation and gene expression. Genes Dev. 2013; 27:985–990. 23603901 10.1101/gad.217778.113 PMC3656328

[150] Mohammad F., Weissmann S., Leblanc B., Pandey D.P., Højfeldt J.W., Comet I., Zheng C., Johansen J.V., Rapin N., Porse B.T. et al. EZH2 is a potential therapeutic target for H3K27M-mutant pediatric gliomas. Nat. Med. 2017; 23:483–492. 28263309 10.1038/nm.4293

[151] Brien G.L., Bressan R.B., Monger C., Gannon D., Lagan E., Doherty A.M., Healy E., Neikes H., Fitzpatrick D.J., Deevy O. et al. Simultaneous disruption of PRC2 and enhancer function underlies histone H3.3-K27M oncogenic activity in human hindbrain neural stem cells. Nat. Genet. 2021; 53:1221–1232. 34294917 10.1038/s41588-021-00897-w

[152] Piunti A., Hashizume R., Morgan M.A., Bartom E.T., Horbinski C.M., Marshall S.A., Rendleman E.J., Ma Q., Takahashi Y., Woodfin A.R. et al. Therapeutic targeting of polycomb and BET bromodomain proteins in diffuse intrinsic pontine gliomas. Nat. Med. 2017; 23:493–500. 28263307 10.1038/nm.4296 PMC5667640

[153] Wiese M., Hamdan F.H., Kubiak K., Diederichs C., Gielen G.H., Nussbaumer G., Carcaboso A.M., Hulleman E., Johnsen S.A., Kramm C.M. Combined treatment with CBP and BET inhibitors reverses inadvertent activation of detrimental super enhancer programs in DIPG cells. Cell Death Dis. 2020; 11:1–13. 32826850 10.1038/s41419-020-02800-7 PMC7442654

[154] Jain S.U., Do T.J., Lund P.J., Rashoff A.Q., Diehl K.L., Cieslik M., Bajic A., Juretic N., Deshmukh S., Venneti S. et al. PFA ependymoma-associated protein EZHIP inhibits PRC2 activity through a H3 K27M-like mechanism. Nat. Commun. 2019; 10:1–14. 31086175 10.1038/s41467-019-09981-6 PMC6513997

[155] Xu B., Konze K.D., Jin J., Wang G.G. Targeting EZH2 and PRC2 dependence as novel anticancer therapy. Exp. Hematol. 2015; 43:698–712. 26027790 10.1016/j.exphem.2015.05.001 PMC4706459

[156] Caganova M., Carrisi C., Varano G., Mainoldi F., Zanardi F., Germain P.-L., George L., Alberghini F., Ferrarini L., Talukder A.K. et al. Germinal center dysregulation by histone methyltransferase EZH2 promotes lymphomagenesis. J. Clin. Invest. 2013; 123:5009–5022. 24200695 10.1172/JCI70626 PMC3859423

[157] Okosun J., Bödör C., Wang J., Araf S., Yang C.-Y., Pan C., Boller S., Cittaro D., Bozek M., Iqbal S. et al. Integrated genomic analysis identifies recurrent mutations and evolution patterns driving the initiation and progression of follicular lymphoma. Nat. Genet. 2013; 46:176–181. 24362818 10.1038/ng.2856 PMC3907271

[158] Knutson S.K., Wigle T.J., Warholic N.M., Sneeringer C.J., Allain C.J., Klaus C.R., Sacks J.D., Raimondi A., Majer C.R., Song J. et al. A selective inhibitor of EZH2 blocks H3K27 methylation and kills mutant lymphoma cells. Nat. Chem. Biol. 2012; 8:890–896. 23023262 10.1038/nchembio.1084

[159] McCabe M.T., Ott H.M., Ganji G., Korenchuk S., Thompson C., Van Aller G.S., Liu Y., Pietra A.D, LaFrance L.V., Mellinger M. et al. EZH2 inhibition as a therapeutic strategy for lymphoma with EZH2-activating mutations. Nature. 2012; 492:108–112. 23051747 10.1038/nature11606

[160] Paret C., Theruvath J., Russo A., Kron B., Malki K.El, Lehmann N., Wingerter A., Neu M.A., Gerhold-Ay A., Wagner W. et al. Activation of the basal cell carcinoma pathway in a patient with CNS HGNET-BCOR diagnosis: consequences for personalized targeted therapy. Oncotarget. 2016; 7:83378–83391. 27825128 10.18632/oncotarget.13092 PMC5347776

[161] Hong S., Cho Y.-W., Yu L.-R., Yu H., Veenstra T.D., Ge K. Identification of JmjC domain-containing UTX and JMJD3 as histone H3 lysine 27 demethylases. Proc. Natl. Acad. Sci. 2007; 104:18439–18444. 18003914 10.1073/pnas.0707292104 PMC2141795

[162] Kim J.-H., Sharma A., Dhar S.S., Lee S.-H., Gu B., Chan C.-H., Lin H.-K., Lee M.G. UTX and MLL4 Coordinately Regulate Transcriptional Programs for Cell Proliferation and Invasiveness in Breast Cancer Cells. Cancer Res. 2014; 74:1705–1717. 24491801 10.1158/0008-5472.CAN-13-1896 PMC3962500

[163] Wang L., Shilatifard A. UTX Mutations in Human Cancer. Cancer Cell. 2019; 35:168. 30753822 10.1016/j.ccell.2019.01.001 PMC6589339

[164] Bracken A.P., Brien G.L., Verrijzer C.P. Dangerous liaisons: interplay between SWI/SNF, NuRD, and Polycomb in chromatin regulation and cancer. Genes Dev. 2019; 33:936–959. 31123059 10.1101/gad.326066.119 PMC6672049

[165] Kadoch C., Crabtree G.R. Mammalian SWI/SNF chromatin remodeling complexes and cancer: Mechanistic insights gained from human genomics. Sci. Adv. 2015; 1:e1500447. 26601204 10.1126/sciadv.1500447 PMC4640607

[166] Wilson B.G., Wang X., Shen X., McKenna E.S., Lemieux M.E., Cho Y.-J., Koellhoffer E.C., Pomeroy S.L., Orkin S.H., Roberts C.W.M. Epigenetic Antagonism between Polycomb and SWI/SNF Complexes during Oncogenic Transformation. Cancer Cell. 2010; 18:316–328. 20951942 10.1016/j.ccr.2010.09.006 PMC2957473

[167] Kim K.H., Roberts C.W.M. Targeting EZH2 in cancer. Nat. Med. 2016; 22:128–134. 26845405 10.1038/nm.4036 PMC4918227

[168] McBride M.J., Pulice J.L., Beird H.C., Ingram D.R., D’Avino A.R., Shern J.F., Charville G.W., Hornick J.L., Nakayama R.T., Garcia-Rivera E.M. et al. The SS18-SSX fusion oncoprotein hijacks BAF complex targeting and function to drive synovial sarcoma. Cancer Cell. 2018; 33:1128–1141. 29861296 10.1016/j.ccell.2018.05.002 PMC6791822

[169] Bitler B.G., Aird K.M., Garipov A., Li H., Amatangelo M., Kossenkov A.V, Schultz D.C., Liu Q., Shih I.-M., Conejo-Garcia J.R. et al. Synthetic lethality by targeting EZH2 methyltransferase activity in ARID1A -mutated cancers. Nat. Med. 2015; 21:231–238. 25686104 10.1038/nm.3799 PMC4352133

[170] Fillmore C.M., Xu C., Desai P.T., Berry J.M., Rowbotham S.P., Lin Y.-J., Zhang H., Marquez V.E., Hammerman P.S., Wong K.-K. et al. EZH2 inhibition sensitizes BRG1 and EGFR mutant lung tumours to TopoII inhibitors. Nature. 2015; 520:239–242. 25629630 10.1038/nature14122 PMC4393352

[171] Wang Y., Chen S.Y., Karnezis A.N., Colborne S., Santos N.D, Lang J.D., Hendricks W.P., Orlando K.A., Yap D., Kommoss F. et al. The histone methyltransferase EZH2 is a therapeutic target in small cell carcinoma of the ovary, hypercalcaemic type. J. Pathol. 2017; 242:371–383. 28444909 10.1002/path.4912 PMC6857704

[172] Kim K.H., Kim W., Howard T.P., Vazquez F., Tsherniak A., Wu J.N., Wang W., Haswell J.R., Walensky L.D., Hahn W.C. et al. SWI/SNF-mutant cancers depend on catalytic and non-catalytic activity of EZH2. Nat. Med. 2015; 21:1491–1496. 26552009 10.1038/nm.3968 PMC4886303

[173] Shi J., Wang E., Zuber J., Rappaport A., Taylor M., Johns C., Lowe S.W., Vakoc C.R. The Polycomb complex PRC2 supports aberrant self-renewal in a mouse model of MLL-AF9;NrasG12D acute myeloid leukemia. Oncogene. 2013; 32:930. 22469984 10.1038/onc.2012.110 PMC4102143

[174] Neff T., Sinha A.U., Kluk M.J., Zhu N., Khattab M.H., Stein L., Xie H., Orkin S.H., Armstrong S.A. Polycomb repressive complex 2 is required for MLL-AF9 leukemia. Proc. Natl. Acad. Sci. 2012; 109:5028–5033. 22396593 10.1073/pnas.1202258109 PMC3324004

[175] Wang S., Denton K.E., Hobbs K.F., Weaver T., McFarlane J.M.B., Connelly K.E., Gignac M.C., Milosevich N., Hof F., Paci I. et al. Optimization of ligands using focused DNA-Encoded Libraries To Develop a Selective, Cell-Permeable CBX8 chromodomain inhibitor. ACS Chem. Biol. 2019; 15:112–131. 31755685 10.1021/acschembio.9b00654 PMC7247616

[176] Mueller D., Bach C., Zeisig D., Garcia-Cuellar M.-P., Monroe S., Sreekumar A., Zhou R., Nesvizhskii A., Chinnaiyan A., Hess J.L. et al. A role for the MLL fusion partner ENL in transcriptional elongation and chromatin modification. Blood. 2007; 110:4445–4454. 17855633 10.1182/blood-2007-05-090514 PMC2234781

[177] Neureiter D., Kiesslich T., Ritter M., Mayr C. Update on the role and therapeutic potential of polycomb repressive complexes in (biliary tract) cancer. Expert Opin Ther Targets. 2017; 22:1–3. 29148857 10.1080/14728222.2018.1406923

[178] Grzenda A., Ordog T., Urrutia R. Polycomb and the Emerging Epigenetics of Pancreatic Cancer. J. Gastrointest. Cancer. 2011; 42:100. 21336826 10.1007/s12029-011-9262-4 PMC3678558

[179] Patil S., Steuber B., Kopp W., Kari V., Urbach L., Wang X., Küffer S., Bohnenberger H., Spyropoulou D., Zhang Z. et al. EZH2 regulates pancreatic cancer subtype identity and tumor progression via transcriptional repression of GATA6. Cancer Res. 2020; 80:4620–4632. 32907838 10.1158/0008-5472.CAN-20-0672

[180] Wang M.-C., Jiao M., Wu T., Jing L., Cui J., Guo H., Tian T., Ruan Z., Wei Y.-C., Jiang L.-L. et al. Polycomb complex protein BMI-1 promotes invasion and metastasis of pancreatic cancer stem cells by activating PI3K/AKT signaling, an ex vivo, in vitro, and in vivo study. Oncotarget. 2016; 7:9586–9599. 26840020 10.18632/oncotarget.7078 PMC4891062

[181] Pasqualucci L., Dalla-Favera R. Genetics of diffuse large b-cell lymphoma. Blood. 2018; 131:2307–2319. 29666115 10.1182/blood-2017-11-764332 PMC5969374

[182] Brach D., Johnston-Blackwell D., Drew A., Lingaraj T., Motwani V., Warholic N.M., Feldman I., Plescia C., Smith J.J., Copeland R.A. et al. EZH2 inhibition by tazemetostat results in altered dependency on B-cell activation signaling in DLBCL. Mol. Cancer Ther. 2017; 16:2586–2597. 28835384 10.1158/1535-7163.MCT-16-0840

[183] Haupt Y., Alexander W.S., Barri G., Klinken S.P., Adams J.M. Novel zinc finger gene implicated as myc collaborator by retrovirally accelerated lymphomagenesis in Eμ-myc transgenic mice. Cell. 1991; 65:753–763. 1904009 10.1016/0092-8674(91)90383-a

[184] Lohuizen M., Frasch M., Wientjens E., Berns A. Sequence similarity between the mammalian bmi-1 proto-oncogene and the Drosophila regulatory genes Psc and Su(z)2. Nature. 1991; 353:353–355. 1922340 10.1038/353353a0

[185] Lessard J., Sauvageau G. Bmi-1 determines the proliferative capacity of normal and leukaemic stem cells. Nature. 2003; 423:255–260. 12714970 10.1038/nature01572

[186] Alkema M.J., Jacobs H., Lohuizen M., Berns A. Perturbation of B and T cell development and predisposition to lymphomagenesis in Eμ Bmi 1 transgenic mice require the Bmi1 RING finger. Oncogene. 1997; 15:899–910. 9285685 10.1038/sj.onc.1201262

[187] Jacobs J.J.L., Scheijen B., Voncken J.W., Kieboom K., Berns A., Van Lohuizen M. Bmi-1 collaborates with c-Myc in tumorigenesis by inhibiting c-Myc- induced apoptosis via INK4a/ARF. Genes Dev. 1999; 13:2678–2690. 10541554 10.1101/gad.13.20.2678 PMC317101

[188] Park I., Qian D., Kiel M., Becker M.W., Pihalja M., Weissman I.L., Morrison S.J., Clarke M.F. Bmi-1 is required for maintenance of adult self-renewing haematopoietic stem cells. Nature. 2003; 423:302–305. 12714971 10.1038/nature01587

[189] Rizo A., Olthof S., Han L., Vellenga E., de Haan G., Schuringa J.J. Repression of BMI1 in normal and leukemic human CD34+ cells impairs self-renewal and induces apoptosis. Blood. 2009; 114:1498–1505. 19556423 10.1182/blood-2009-03-209734

[190] Béguelin W., Teater M., Gearhart M.D., Calvo Fernández M.T., Goldstein R.L., Cárdenas M.G., Hatzi K., Rosen M., Shen H., Corcoran C.M. et al. EZH2 and BCL6 Cooperate to Assemble CBX8-BCOR complex to repress bivalent promoters, mediate germinal center formation and lymphomagenesis. Cancer Cell. 2016; 30:197–213. 27505670 10.1016/j.ccell.2016.07.006 PMC5000552

[191] Tara S., Isshiki Y., Nakajima-Takagi Y., Oshima M., Aoyama K., Tanaka T., Shinoda D., Koide S., Saraya A., Miyagi S. et al. Bcor insufficiency promotes initiation and progression of myelodysplastic syndrome. Blood. 2018; 132:2470–2483. 30228234 10.1182/blood-2018-01-827964 PMC6450057

[192] Słabicki M., Yoon H., Koeppel J., Nitsch L., Roy Burman S.S., Di Genua C., Donovan K.A., Sperling A.S., Hunkeler M., Tsai J.M. et al. Small-molecule-induced polymerization triggers degradation of BCL6. Nature. 2020; 588:164–168. 33208943 10.1038/s41586-020-2925-1 PMC7816212

[193] Kerres N., Steurer S., Schlager S., Bader G., Berger H., Caligiuri M., Dank C., Engen J.R., Ettmayer P., Fischerauer B. et al. Chemically Induced Degradation of the Oncogenic Transcription Factor BCL6. Cell Rep. 2017; 20:2860–2875. 28930682 10.1016/j.celrep.2017.08.081

[194] McCoull W., Cheung T., Anderson E., Barton P., Burgess J., Byth K., Cao Q., Castaldi M.P., Chen H., Chiarparin E. et al. Development of a Novel B-Cell Lymphoma 6 (BCL6) PROTAC to Provide Insight into Small Molecule Targeting of BCL6. ACS Chem. Biol. 2018; 13:3131–3141. 30335946 10.1021/acschembio.8b00698

[195] McAllister T.E., England K.S., Hopkinson R.J., Brennan P.E., Kawamura A., Schofield C.J. Recent Progress in Histone Demethylase Inhibitors. J. Med. Chem. 2016; 59:1308–1329. 26710088 10.1021/acs.jmedchem.5b01758

[196] Lee J., Kotliarova S., Kotliarov Y., Li A., Su Q., Donin N.M., Pastorino S., Purow B.W., Christopher N., Zhang W. et al. Tumor stem cells derived from glioblastomas cultured in bFGF and EGF more closely mirror the phenotype and genotype of primary tumors than do serum-cultured cell lines. Cancer Cell. 2006; 9:391–403. 16697959 10.1016/j.ccr.2006.03.030

[197] Shi B., Liang J., Yang X., Wang Y., Zhao Y., Wu H., Sun L., Zhang Y., Chen Y., Li R. et al. Integration of Estrogen and Wnt Signaling Circuits by the Polycomb Group Protein EZH2 in Breast Cancer Cells. Mol. Cell. Biol. 2007; 27:5105–5119. 17502350 10.1128/MCB.00162-07 PMC1951944

[198] Su I.H., Dobenecker M.W., Dickinson E., Oser M., Basavaraj A., Marqueron R., Viale A., Reinberg D., Wülfing C., Tarakhovsky A. Polycomb group protein Ezh2 controls actin polymerization and cell signaling. Cell. 2005; 121:425–436. 15882624 10.1016/j.cell.2005.02.029

[199] Ma A., Stratikopoulos E., Park K.S., Wei J., Martin T.C., Yang X., Schwarz M., Leshchenko V., Rialdi A., Dale B. et al. Discovery of a first-in-class EZH2 selective degrader. Nat. Chem. Biol. 2020; 16:214–222. 31819273 10.1038/s41589-019-0421-4 PMC6982609

[200] Gao S., Wang S.-Y., Zhang X.-D., Wu H., Pang D. Low expression of the polycomb protein RING1 predicts poor prognosis in human breast cancer. Front. Oncol. 2021; 10:3393. 10.3389/fonc.2020.618768 PMC7900562 33634028

[201] Zhang C.Z., Chen S.L., Wang C.H., He Y.F., Yang X., Xie D., Yun J.P. CBX8 exhibits oncogenic activity via AKT/b-catenin activation in hepatocellular carcinoma. Cancer Res. 2018; 78:51–63. 29066512 10.1158/0008-5472.CAN-17-0700

[202] Chung C.-Y., Sun Z., Mullokandov G., Bosch A., Qadeer Z.A., Cihan E., Rapp Z., Parsons R., Aguirre-Ghiso J.A., Farias E.F. et al. Cbx8 Acts Non-canonically with Wdr5 to Promote Mammary Tumorigenesis. Cell Rep. 2016; 16:472–486. 27346354 10.1016/j.celrep.2016.06.002 PMC4972459

[203] Zheng S., Lv P., Su J., Miao K., Xu H., Li M. Overexpression of CBX2 in breast cancer promotes tumor progression through the PI3K/AKT signaling pathway. Am. J. Transl. Res. 2019; 11:1668–1682. 30972192 PMC6456535

[204] Clermont P.L., Crea F., Chiang Y.T., Lin D., Zhang A., Wang J.Z.L., Parolia A., Wu R., Xue H., Wang Y. et al. Identification of the epigenetic reader CBX2 as a potential drug target in advanced prostate cancer. Clin. Epigenetics. 2016; 8:1–14. 26877821 10.1186/s13148-016-0182-9 PMC4751702

[205] Wheeler L.J., Watson Z.L., Qamar L., Yamamoto T.M., Post M.D., Berning A.A., Spillman M.A., Behbakht K., Bitler B.G. CBX2 identified as driver of anoikis escape and dissemination in high grade serous ovarian cancer. Oncogenesis. 2018; 7:1–14. 30478317 10.1038/s41389-018-0103-1 PMC6255906

[206] Pallante P., Federico A., Berlingieri M.T., Bianco M., Ferraro A., Forzati F., Iaccarino A., Russo M., Pierantoni G.M., Leone V. et al. Loss of the CBX7 Gene Expression Correlates with a Highly Malignant Phenotype in Thyroid Cancer. Cancer Res. 2008; 68:6770–6778. 18701502 10.1158/0008-5472.CAN-08-0695

[207] Forzati F., Federico A., Pallante P., Abbate A., Esposito F., Malapelle U., Sepe R., Palma G., Troncone G., Scarfò M. et al. CBX7 is a tumor suppressor in mice and humans. J. Clin. Invest. 2013; 123:934–934. 10.1172/JCI58620 PMC3266782 22214847

[208] Li G., Warden C., Zou Z., Neman J., Krueger J.S., Jain A., Jandial R., Chen M. Altered expression of polycomb group genes in glioblastoma multiforme. PLoS One. 2013; 8:e80970. 24260522 10.1371/journal.pone.0080970 PMC3829908

[209] Huang Z., Yan Y., Zhu Z., Liu J., He X., Dalangood S., Li M., Tan M., Cai J., Tang P. et al. CBX7 suppresses urinary bladder cancer progression via modulating AKR1B10–ERK signaling. Cell Death Dis. 2021; 12:1–15. 34035231 10.1038/s41419-021-03819-0 PMC8149849

[210] Li R., Yan Q., Tian P., Wang Y., Wang J., Tao N., Ning L., Lin X., Ding L., Liu J. et al. CBX7 inhibits cell growth and motility and induces apoptosis in cervical cancer cells. Mol. Ther. - Oncolytics. 2019; 15:108–116. 31709304 10.1016/j.omto.2019.09.002 PMC6834976

[211] Deng H., Guan X., Gong L., Zeng J., Zhang H., Chen M.Y., Li G. CBX6 is negatively regulated by EZH2 and plays a potential tumor suppressor role in breast cancer. Sci. Rep. 2019; 9:1–13. 30655550 10.1038/s41598-018-36560-4 PMC6336801

[212] Scott C.L., Gil J., Hernando E., Teruya-Feldstein J., Narita M., Martínez D., Visakorpi T., Mu D., Cordon-Cardo C., Peters G. et al. Role of the chromobox protein CBX7 in lymphomagenesis. Proc. Natl. Acad. Sci. 2007; 104:5389–5394. 17374722 10.1073/pnas.0608721104 PMC1828941

[213] Bernard D., Martinez-Leal J.F., Rizzo S., Martinez D., Hudson D., Visakorpi T., Peters G., Carnero A., Beach D., Gil J. CBX7 controls the growth of normal and tumor-derived prostate cells by repressing the Ink4a/Arf locus. Oncogene. 2005; 24:5543–5551. 15897876 10.1038/sj.onc.1208735

[214] Zheng H., Jiang W.H., Tian T., Tan H.S., Chen Y., Qiao G.L., Han J., Huang S.Y., Yang Y., Li S. et al. CBX6 overexpression contributes to tumor progression and is predictive of a poor prognosis in hepatocellular carcinoma. Oncotarget. 2017; 8:18872–18884. 28122351 10.18632/oncotarget.14770 PMC5386654

[215] Plys A.J., Davis C.P., Kim J., Rizki G., Keenen M.M., Marr S.K., Kingston R.E. Phase separation of polycomb-repressive complex 1 is governed by a charged disordered region of CBX2. Genes Dev. 2019; 33:799–813. 31171700 10.1101/gad.326488.119 PMC6601514

[216] Kundu S., Ji F., Sunwoo H., Jain G., Lee J.T., Sadreyev R.I., Dekker J., Kingston R.E. Polycomb Repressive Complex 1 Generates Discrete Compacted Domains that Change during Differentiation. Mol. Cell. 2017; 65:432–446. 28157505 10.1016/j.molcel.2017.01.009 PMC5421375

[217] Varambally S., Dhanasekaran S.M., Zhou M., Barrette T.R., Kumar-Sinha C., Sanda M.G., Ghosh D., Pienta K.J., Sewalt R.G.A.B., Rubin M.A. et al. The polycomb group protein EZH2 is involved in progression of prostate cancer. Nature. 2002; 419:624–629. 12374981 10.1038/nature01075

[218] van Leenders G.J.L.H., Dukers D., Hessels D., van den Kieboom S.W.M., Hulsbergen C.A., Witjes J.A., Otte A.P., Meijer C.J., Raaphorst F.M. Polycomb-Group Oncogenes EZH2, BMI1, and RING1 Are Overexpressed in Prostate Cancer With Adverse Pathologic and Clinical Features. Eur. Urol. 2007; 52:455–463. 17134822 10.1016/j.eururo.2006.11.020

[219] Clermont P.L., Lin D., Crea F., Wu R., Xue H., Wang Y., Thu K.L., Lam W.L., Collins C.C., Wang Y. et al. Polycomb-mediated silencing in neuroendocrine prostate cancer. Clin. Epigenetics. 2015; 7:1–13. 25859291 10.1186/s13148-015-0074-4 PMC4391120

[220] Pickl J.M.A., Tichy D., Kuryshev V.Y., Tolstov Y., Falkenstein M., Schüler J., Reidenbach D., Hotz-Wagenblatt A., Kristiansen G., Roth W. et al. Ago-RIP-Seq identifies Polycomb repressive complex I member CBX7 as a major target of miR-375 in prostate cancer progression. Oncotarget. 2016; 7:59589–59603. 27449098 10.18632/oncotarget.10729 PMC5312160

[221] Zacharopoulou N., Tsapara A., Kallergi G., Schmid E., Tsichlis P.N., Kampranis S.C., Stournaras C. The epigenetic factor KDM2B regulates cell adhesion, small rho GTPases, actin cytoskeleton and migration in prostate cancer cells. Biochim. Biophys. Acta - Mol. Cell Res. 2018; 1865:587–597. 29408056 10.1016/j.bbamcr.2018.01.009

[222] Liu Q., Wang G., Li Q., Jiang W., Kim J.-S., Wang R., Zhu S., Wang X., Yan L., Yi Y. et al. Polycomb group proteins EZH2 and EED directly regulate androgen receptor in advanced prostate cancer. Int. J. Cancer. 2019; 145:415–426. 30628724 10.1002/ijc.32118 PMC7423571

[223] Jain P., Ballare C., Blanco E., Vizan P., Di Croce L. PHF19 mediated regulation of proliferation and invasiveness in prostate cancer cells. Elife. 2020; 9:e51373. 32155117 10.7554/eLife.51373 PMC7064337

[224] Zhu J., Jin L., Zhang A., Gao P., Dai G., Xu M., Xu L., Yang D. Coexpression analysis of the EZH2 gene using the cancer genome atlas and oncomine databases identifies coexpressed genes involved in biological networks in breast cancer, glioblastoma, and prostate cancer. Med. Sci. Monit. 2020; 26:e922346. 32595202 10.12659/MSM.922346 PMC7320634

[225] Xu K., Wu Z.J., Groner A.C., He H.H., Cai C., Lis R.T., Wu X., Stack E.C., Loda M., Liu T. et al. EZH2 oncogenic activity in castration-resistant prostate cancer cells is polycomb-independent. Science. 2012; 338:1465–1469. 23239736 10.1126/science.1227604 PMC3625962

[226] Wang S., Alpsoy A., Sood S., Ordonez-Rubiano S.C., Dhiman A., Sun Y., Jiao G., Krusemark C.J., Dykhuizen E.C. A potent, selective CBX2 chromodomain ligand and its cellular activity during prostate cancer neuroendocrine differentiation. ChemBioChem. 2021; 22:2335–2344. 33950564 10.1002/cbic.202100118 PMC8358665

[227] Dardenne E., Beltran H., Benelli M., Gayvert K., Berger A., Puca L., Cyrta J., Sboner A., Noorzad Z., MacDonald T. et al. N-Myc induces an EZH2-Mediated transcriptional program driving neuroendocrine prostate cancer. Cancer Cell. 2016; 30:563–577. 27728805 10.1016/j.ccell.2016.09.005 PMC5540451

[228] Bai Y., Zhang Z., Cheng L., Wang R., Chen X., Kong Y., Feng F., Ahmad N., Li L., Liu X. Inhibition of enhancer of zeste homolog 2 (EZH2) overcomes enzalutamide resistance in castration-resistant prostate cancer. J. Biol. Chem. 2019; 294:9911–9923. 31085587 10.1074/jbc.RA119.008152 PMC6597805

[229] Li Q., Liu K.Y., Liu Q., Wang G., Jiang W., Meng Q., Yi Y., Yang Y., Wang R., Zhu S. et al. Antihistamine drug ebastine inhibits cancer growth by targeting polycomb group protein EZH2. Mol. Cancer Ther. 2020; 19:2023–2033. 32855270 10.1158/1535-7163.MCT-20-0250 PMC7541747

[230] Zhu S., Zhao D., Yan L., Jiang W., Kim J.-S., Gu B., Liu Q., Wang R., Xia B., Zhao J.C. et al. BMI1 regulates androgen receptor in prostate cancer independently of the polycomb repressive complex 1. Nat. Commun. 2018; 9:1–13. 29402932 10.1038/s41467-018-02863-3 PMC5799368

[231] Zhu S., Zhao D., Li C., Li Q., Jiang W., Liu Q., Wang R., Fazli L., Li Y., Zhang L. et al. BMI1 is directly regulated by androgen receptor to promote castration-resistance in prostate cancer. Oncogene. 2019; 39:17–29. 31462713 10.1038/s41388-019-0966-4 PMC7386438

[232] Umbreen S., Banday M.M., Jamroze A., Mansini A.P., Ganaie A.A., Ferrari M.G., Maqbool R., Beigh F.H., Murugan P., Morrissey C. et al. COMMD3:BMI1 Fusion and COMMD3 Protein Regulate C-MYC Transcription: Novel therapeutic target for metastatic prostate cancer. Mol. Cancer Ther. 2019; 18:2111–2123. 31467179 10.1158/1535-7163.MCT-19-0150

[233] Su W., Han H.H., Wang Y., Zhang B., Zhou B., Cheng Y., Rumandla A., Gurrapu S., Chakraborty G., Su J. et al. The polycomb repressor complex 1 Drives Double-Negative prostate cancer metastasis by coordinating stemness and immune suppression. Cancer Cell. 2019; 36:139–155. 31327655 10.1016/j.ccell.2019.06.009 PMC7210785

[234] Yi Y., Hsieh I.-Y., Huang X., Li J., Zhao W. Glioblastoma stem-like cells: characteristics, microenvironment, and therapy. Front. Pharmacol. 2016; 7:477. 28003805 10.3389/fphar.2016.00477 PMC5141588

[235] Chen J., Li Y., Yu T.-S., McKay R.M., Burns D.K., Kernie S.G., Parada L.F. A restricted cell population propagates glioblastoma growth after chemotherapy. Nature. 2012; 488:522–526. 22854781 10.1038/nature11287 PMC3427400

[236] Singh S.K., Hawkins C., Clarke I.D., Squire J.A., Bayani J., Hide T., Henkelman R.M., Cusimano M.D., Dirks P.B. Identification of human brain tumour initiating cells. Nature. 2004; 432:396–401. 15549107 10.1038/nature03128

[237] Abdouh M., Facchino S., Chatoo W., Balasingam V., Ferreira J., Bernier G. BMI1 sustains human glioblastoma multiforme stem cell renewal. J. Neurosci. 2009; 29:8884–8896. 19605626 10.1523/JNEUROSCI.0968-09.2009 PMC6665439

[238] Kreso A., Galen P., Pedley N.M., Lima-Fernandes E., Frelin C., Davis T., Cao L., Baiazitov R., Du W., Sydorenko N. et al. Self-renewal as a therapeutic target in human colorectal cancer. Nat. Med. 2013; 20:29–36. 24292392 10.1038/nm.3418

[239] Martin M.C., Zeng G., Yu J., Schiltz G.E. Small molecule approaches for targeting the polycomb repressive complex 2 (PRC2) in Cancer. J. Med. Chem. 2020; 63:15344–15370. 33283516 10.1021/acs.jmedchem.0c01344

[240] Tan J., Yang X., Zhuang L., Jiang X., Chen W., Puay L.L., Karuturi R.K.M., Tan P.B.O., Liu E.T., Yu Q. Pharmacologic disruption of polycomb-repressive complex 2-mediated gene repression selectively induces apoptosis in cancer cells. Genes Dev. 2007; 21:1050–1063. 17437993 10.1101/gad.1524107 PMC1855231

[241] Verma S.K., Tian X., Lafrance L.V., Duquenne C., Suarez D.P., Newlander K.A., Romeril S.P., Burgess J.L., Grant S.W., Brackley J.A. et al. Identification of potent, selective, cell-Active inhibitors of the histone lysine methyltransferase EZH2. ACS Med. Chem. Lett. 2012; 3:1091–1096. 24900432 10.1021/ml3003346 PMC4025676

[242] Shen J.K., Cote G.M., Gao Y., Choy E., Mankin H.J., Hornicek F.J., Duan Z. Targeting EZH2-mediated methylation of H3K27 inhibits proliferation and migration of Synovial Sarcoma in vitro. Sci. Rep. 2016; 6:1–10. 27125524 10.1038/srep25239 PMC4850444

[243] Knutson S.K., Kawano S., Minoshima Y., Warholic N.M., Huang K.-C., Xiao Y., Kadowaki T., Uesugi M., Kuznetsov G., Kumar N. et al. Selective Inhibition of EZH2 by EPZ-6438 Leads to Potent Antitumor Activity in EZH2-Mutant Non-Hodgkin Lymphoma. Mol. Cancer Ther. 2014; 13:842–854. 24563539 10.1158/1535-7163.MCT-13-0773

[244] Morschhauser F., Tilly H., Chaidos A., McKay P., Phillips T., Assouline S., Batlevi C.L., Campbell P., Ribrag V., Damaj G.L. et al. Tazemetostat for patients with relapsed or refractory follicular lymphoma: an open-label, single-arm, multicentre, phase 2 trial. Lancet Oncol. 2020; 21:1433–1442. 33035457 10.1016/S1470-2045(20)30441-1 PMC8427481

[245] Italiano A., Soria J.-C., Toulmonde M., Michot J.-M., Lucchesi C., Varga A., Coindre J.-M., Blakemore S.J., Clawson A., Suttle B. et al. Tazemetostat, an EZH2 inhibitor, in relapsed or refractory B-cell non-Hodgkin lymphoma and advanced solid tumours: a first-in-human, open-label, phase 1 study. Lancet Oncol. 2018; 19:649–659. 29650362 10.1016/S1470-2045(18)30145-1

[246] Gounder M., Schöffski P., Jones R.L., Agulnik M., Cote G.M., Villalobos V.M., Attia S., Chugh R., Chen T.W.W., Jahan T. et al. Tazemetostat in advanced epithelioid sarcoma with loss of INI1/SMARCB1: an international, open-label, phase 2 basket study. Lancet Oncol. 2020; 21:1423–1432. 33035459 10.1016/S1470-2045(20)30451-4

[247] Campbell J.E., Kuntz K.W., Knutson S.K., Warholic N.M., Keilhack H., Wigle T.J., Raimondi A., Klaus C.R., Rioux N., Yokoi A. et al. EPZ011989, A potent, orally-available EZH2 inhibitor with robust in vivo activity. ACS Med. Chem. Lett. 2015; 6:491–495. 26005520 10.1021/acsmedchemlett.5b00037 PMC4434464

[248] Qi W., Chan H., Teng L., Li L., Chuai S., Zhang R., Zeng J., Li M., Fan H., Lin Y. et al. Selective inhibition of Ezh2 by a small molecule inhibitor blocks tumor cells proliferation. Proc. Natl. Acad. Sci. 2012; 109:21360–21365. 23236167 10.1073/pnas.1210371110 PMC3535655

[249] Konze K.D., Ma A., Li F., Barsyte-Lovejoy D., Parton T., MacNevin C.J., Liu F., Gao C., Huang X.-P., Kuznetsova E. et al. An orally bioavailable chemical probe of the lysine methyltransferases EZH2 and EZH1. ACS Chem. Biol. 2013; 8:1324–1334. 23614352 10.1021/cb400133j PMC3773059

[250] Xu B., On D.M., Ma A., Parton T., Konze K.D., Pattenden S.G., Allison D.F., Cai L., Rockowitz S., Liu S. et al. Selective inhibition of EZH2 and EZH1 enzymatic activity by a small molecule suppresses MLL-rearranged leukemia. Blood. 2015; 125:346–357. 25395428 10.1182/blood-2014-06-581082 PMC4287641

[251] Bradley W.D., Arora S., Busby J., Balasubramanian S., Gehling V.S., Nasveschuk C.G., Vaswani R.G., Yuan C.C., Hatton C., Zhao F. et al. EZH2 inhibitor efficacy in Non-Hodgkin's lymphoma does not require suppression of H3K27 monomethylation. Chem. Biol. 2014; 21:1463–1475. 25457180 10.1016/j.chembiol.2014.09.017

[252] Vaswani R.G., Gehling V.S., Dakin L.A., Cook A.S., Nasveschuk C.G., Duplessis M., Iyer P., Balasubramanian S., Zhao F., Good A.C. et al. Identification of (R)-N-((4-Methoxy-6-methyl-2-oxo-1,2-dihydropyridin-3-yl)methyl)-2-methyl-1-(1-(1-(2,2,2-trifluoroethyl)piperidin-4-yl)ethyl)-1H-indole-3-carboxamide (CPI-1205), a Potent and Selective Inhibitor of Histone Methyltransferase EZH2, Suitable for Phase I Clinical Trials for B-Cell Lymphomas. J. Med. Chem. 2016; 59:9928–9941. 27739677 10.1021/acs.jmedchem.6b01315 PMC5451150

[253] Kung P.-P., Bingham P., Brooun A., Collins M., Deng Y.-L., Dinh D., Fan C., Gajiwala K.S., Grantner R., Gukasyan H.J. et al. Optimization of Orally Bioavailable Enhancer of Zeste Homolog 2 (EZH2) Inhibitors Using Ligand and Property-Based Design Strategies: Identification of Development Candidate (R)-5,8-Dichloro-7-(methoxy(oxetan-3-yl)methyl)-2-((4-methoxy-6-methyl-2-oxo-1,2-dihydropyridin-3-yl)methyl)-3,4-dihydroisoquinolin-1(2H)-one (PF-06821497). J. Med. Chem. 2017; 61:650–665. 29211475 10.1021/acs.jmedchem.7b01375

[254] Honma D., Kanno O., Watanabe J., Kinoshita J., Hirasawa M., Nosaka E., Shiroishi M., Takizawa T., Yasumatsu I., Horiuchi T. et al. Novel orally bioavailable EZH1/2 dual inhibitors with greater antitumor efficacy than an EZH2 selective inhibitor. Cancer Sci. 2017; 108:2069–2078. 28741798 10.1111/cas.13326 PMC5623739

[255] Fujita S., Honma D., Adachi N., Araki K., Takamatsu E., Katsumoto T., Yamagata K., Akashi K., Aoyama K., Iwama A. et al. Dual inhibition of EZH1/2 breaks the quiescence of leukemia stem cells in acute myeloid leukemia. Leukemia. 2017; 32:855–864. 28951561 10.1038/leu.2017.300

[256] Neklesa T.K., Crews C.M. Chemical biology: Greasy tags for protein removal. Nature. 2012; 487:308–309. 22810693 10.1038/487308a

[257] Burslem G.M., Crews C.M. Small-molecule modulation of protein homeostasis. Chem. Rev. 2017; 117:11269–11301. 28777566 10.1021/acs.chemrev.7b00077

[258] Kong X., Chen L., Jiao L., Jiang X., Lian F., Lu J., Zhu K., Du D., Liu J., Ding H. et al. Astemizole arrests the proliferation of cancer cells by disrupting the EZH2-EED interaction of polycomb repressive complex 2. J. Med. Chem. 2014; 57:9512–9521. 25369470 10.1021/jm501230c

[259] Kim W., Bird G.H., Neff T., Guo G., Kerenyi M.A., Walensky L.D., Orkin S.H. Targeted disruption of the EZH2-EED complex inhibits EZH2-dependent cancer. Nat. Chem. Biol. 2013; 9:643–650. 23974116 10.1038/nchembio.1331 PMC3778130

[260] Du D., Xu D., Zhu L., Stazi G., Zwergel C., Liu Y., Luo Z., Li Y., Zhang Y., Zhu K. et al. Structure-Guided Development of Small-Molecule PRC2 Inhibitors Targeting EZH2–EED Interaction. J. Med. Chem. 2021; 64:8194–8207. 34077206 10.1021/acs.jmedchem.0c02261

[261] He Y., Selvaraju S., Curtin M.L., Jakob C.G., Zhu H., Comess K.M., Shaw B., The J., Lima-Fernandes E., Szewczyk M.M. et al. The EED protein-protein interaction inhibitor A-395 inactivates the PRC2 complex. Nat. Chem. Biol. 2017; 13:389–395. 28135237 10.1038/nchembio.2306

[262] Curtin M.L., Pliushchev M.A., Li H.Q., Torrent M., Dietrich J.D., Jakob C.G., Zhu H., Zhao H., Wang Y., Ji Z. et al. SAR of amino pyrrolidines as potent and novel protein-protein interaction inhibitors of the PRC2 complex through EED binding. Bioorganic Med. Chem. Lett. 2017; 27:1576–1583. 10.1016/j.bmcl.2017.02.030 28254486

[263] Qi W., Zhao K., Gu J., Huang Y., Wang Y., Zhang H., Zhang M., Zhang J., Yu Z., Li L. et al. An allosteric PRC2 inhibitor targeting the H3K27me3 binding pocket of EED. Nat. Chem. Biol. 2017; 13:381–388. 28135235 10.1038/nchembio.2304

[264] Huang Y., Zhang J., Yu Z., Zhang H., Wang Y., Lingel A., Qi W., Gu J., Zhao K., Shultz M.D. et al. Discovery of First-in-Class, potent, and orally bioavailable embryonic ectoderm development (EED) inhibitor with robust anticancer efficacy. J. Med. Chem. 2017; 60:2215–2226. 28092155 10.1021/acs.jmedchem.6b01576

[265] US20160176882A1 - Triazolopyrimidine compounds and uses thereof - Google Patents.

[266] Barnash K.D., The J., Norris-Drouin J.L., Cholensky S.H., Worley B.M., Li F., Stuckey J.I., Brown P.J., Vedadi M., Arrowsmith C.H. et al. Discovery of peptidomimetic ligands of EED as allosteric inhibitors of PRC2. ACS Comb. Sci. 2017; 19:161–172. 28165227 10.1021/acscombsci.6b00174 PMC5376495

[267] Lingel A., Sendzik M., Huang Y., Shultz M.D., Cantwell J., Dillon M.P., Fu X., Fuller J., Gabriel T., Gu J. et al. Structure-guided design of EED binders allosterically inhibiting the epigenetic polycomb repressive complex 2 (PRC2) methyltransferase. J. Med. Chem. 2017; 60:415–427. 27992714 10.1021/acs.jmedchem.6b01473

[268] Potjewyd F., Turner A.-M.W., Beri J., Rectenwald J.M., Norris-Drouin J.L., Cholensky S.H., Margolis D.M., Pearce K.H., Herring L.E., James L.I. Degradation of Polycomb Repressive Complex 2 with an EED-Targeted Bivalent Chemical Degrader. Cell Chem. Biol. 2020; 27:47–56. 31831267 10.1016/j.chembiol.2019.11.006 PMC7004250

[269] Hsu J.H., Rasmusson T., Robinson J., Pachl F., Read J., Kawatkar S., Donovan D.H.O., Bagal S., Code E., Rawlins P. et al. EED-Targeted PROTACs Degrade EED, EZH2, and SUZ12 in the PRC2 Complex. Cell Chem. Biol. 2020; 27:41–46. 31786184 10.1016/j.chembiol.2019.11.004

[270] Suh J.L., Barnash K.D., Abramyan T.M., Li F., The J., Engelberg I.A., Vedadi M., Brown P.J., Kireev D.B., Arrowsmith C.H. et al. Discovery of selective activators of PRC2 mutant EED-I363M. Sci. Rep. 2019; 9:1–10. 31024026 10.1038/s41598-019-43005-z PMC6484020

[271] Kadoch C., Copeland R.A., Keilhack H. PRC2 and SWI/SNF chromatin remodeling complexes in health and disease. Biochemistry. 2016; 55:1600–1614. 26836503 10.1021/acs.biochem.5b01191

[272] Kogure M., Takawa M., Saloura V., Sone K., Piao L., Ueda K., Ibrahim R., Tsunoda T., Sugiyama M., Atomi Y. et al. The oncogenic polycomb histone methyltransferase EZH2Methylates Lysine 120 on Histone H2B and competes ubiquitination. Neoplasia. 2013; 15:1251–IN10. 24339737 10.1593/neo.131436 PMC3858897

[273] Gunawan M., Venkatesan N., Loh J.T., Wong J.F., Berger H., Neo W.H., Li L.Y.J., La Win M.K., Yau Y.H., Guo T. et al. The methyltransferase Ezh2 controls cell adhesion and migration through direct methylation of the extranuclear regulatory protein talin. Nat. Immunol. 2015; 16:505–516. 25751747 10.1038/ni.3125

[274] Kim E., Kim M., Woo D.H., Shin Y., Shin J., Chang N., Oh Y.T., Kim H., Rheey J., Nakano I. et al. Phosphorylation of EZH2 Activates STAT3 Signaling via STAT3 methylation and promotes tumorigenicity of glioblastoma Stem-like Cells. Cancer Cell. 2013; 23:839–852. 23684459 10.1016/j.ccr.2013.04.008 PMC4109796

[275] Ismail I.H., McDonald D., Strickfaden H., Xu Z., Hendzel M.J. A small molecule inhibitor of polycomb repressive complex 1 inhibits ubiquitin signaling at DNA double-strand breaks. J. Biol. Chem. 2013; 288:26944–26954. 23902761 10.1074/jbc.M113.461699 PMC3772243

[276] Shukla S., Ying W., Gray F., Yao Y., Simes M.L., Zhao Q., Miao H., Cho H.J., González-Alonso P., Winkler A. et al. Small-molecule inhibitors targeting Polycomb repressive complex 1 RING domain. Nat. Chem. Biol. 2021; 17:784–793. 34155404 10.1038/s41589-021-00815-5 PMC8238916

[277] Kim M.J., Cao L., Sheedy J., Risher N., Dumble M., Lee C.-S., Sydorenko N., Baiazitov R., Du W., Moon Y.-C. et al. Abstract 5517: PTC596-induced Bmi1 hyper-phosphorylation via Cdk1/2 activation resulting in tumor stem cell depletion. Cancer Res. 2014; 74:5517–5517.

[278] Wang J., Xing Y., Wang Y., He Y., Wang L., Peng S., Yang L., Xie J., Li X., Qiu W. et al. A novel BMI-1 inhibitor QW24 for the treatment of stem-like colorectal cancer. J. Exp. Clin. Cancer Res. 2019; 38:1–14. 31640758 10.1186/s13046-019-1392-8 PMC6805542

[279] Gil J., Bernard D., Martínez D., Beach D. Polycomb CBX7 has a unifying role in cellular lifespan. Nat. Cell Biol. 2004; 6:67–72. 14647293 10.1038/ncb1077

[280] Jung J., Buisman S.C., Weersing E., Dethmers-Ausema A., Zwart E., Schepers H., Dekker M.R., Lazare S.S., Hammerl F., Skokova Y. et al. CBX7 Induces Self-Renewal of human normal and malignant hematopoietic stem and progenitor cells by canonical and Non-canonical interactions. Cell Rep. 2019; 26:1906–1918. 30759399 10.1016/j.celrep.2019.01.050

[281] Simhadri C., Daze K.D., Douglas S.F., Quon T.T.H., Dev A., Gignac M.C., Peng F., Heller M., Boulanger M.J., Wulff J.E. et al. Chromodomain antagonists that target the polycomb-group methyllysine reader protein chromobox homolog 7 (CBX7). J. Med. Chem. 2014; 57:2874–2883. 24625057 10.1021/jm401487x

[282] Stuckey J.I., Dickson B.M., Cheng N., Liu Y., Norris J.L., Cholensky S.H., Tempel W., Qin S., Huber K.G., Sagum C. et al. A cellular chemical probe targeting the chromodomains of Polycomb repressive complex 1. Nat. Chem. Biol. 2016; 12:180–187. 26807715 10.1038/nchembio.2007 PMC4755828

[283] Ren C., Morohashi K., Walsh M., Correspondence M.-M.Z. Small-Molecule Modulators of Methyl-Lysine Binding for the CBX7 Chromodomain Chemical inhibition of CBX7 chromodomain derepresses p16/CDKN2A in PC3 cells. Chem. Biol. 2015; 22:161–168. 25660273 10.1016/j.chembiol.2014.11.021 PMC4336573

[284] Ren C., Smith S.G., Yap K., Li S., Li J., Mezei M., Rodriguez Y., Vincek A., Aguilo F., Walsh M.J. et al. Structure-Guided discovery of selective antagonists for the chromodomain of polycomb repressive protein CBX7. ACS Med. Chem. Lett. 2016; 7:601–605. 27326334 10.1021/acsmedchemlett.6b00042 PMC4904266

[285] Simhadri C., Daze K.D., Douglas S.F., Milosevich N., Monjas L., Dev A., Brown T.M., Hirsch A.K.H., Wulff J.E., Hof F. Rational adaptation of L3MBTL1 inhibitors to create Small-Molecule Cbx7 antagonists. Chem. Med. Chem. 2019; 14:1444–1456. 31254321 10.1002/cmdc.201900021

[286] Lamb K.N., Bsteh D., Dishman S.N., Moussa H.F., Fan H., Stuckey J.I., Norris J.L., Cholensky S.H., Li D., Wang J. et al. Discovery and characterization of a cellular potent positive allosteric modulator of the polycomb repressive complex 1 chromodomain, CBX7. Cell Chem. Biol. 2019; 26:1365–1379. 31422906 10.1016/j.chembiol.2019.07.013 PMC6800648

[287] Milosevich N., Gignac M.C., McFarlane J., Simhadri C., Horvath S., Daze K.D., Croft C.S., Dheri A., Quon T.T.H., Douglas S.F. et al. Selective inhibition of CBX6: A methyllysine reader protein in the polycomb family. ACS Med. Chem. Lett. 2015; 7:139–144. 26985288 10.1021/acsmedchemlett.5b00378 PMC4753544

[288] Milosevich N., Wilson C.R., Brown T.M., Alpsoy A., Wang S., Connelly K.E., Sinclair K.A.D., Ponio F.R., Hof R., Dykhuizen E.C. et al. Polycomb paralog chromodomain inhibitors active against both CBX6 and CBX8. ChemMedChem. 2021; 16:1–9. 10.1002/cmdc.202100262 PMC8497432 34174168

[289] Denton K.E., Wang S., Gignac M.C., Milosevich N., Hof F., Dykhuizen E.C., Krusemark C.J. Robustness of in vitro selection assays of DNA-Encoded peptidomimetic ligands to CBX7 and CBX8. SLAS Discov. 2018; 23:417–428. 29309209 10.1177/2472555217750871 PMC5962403

[290] Suh J.L., Bsteh D., Si Y., Hart B., Weaver T.M., Pribitzer C., Lau R., Soni S., Ogana H., Rectenwald J.M. et al. Reprogramming CBX8-PRC1 function with a positive allosteric modulator. 2021; bioRxiv doi23 February 2021, preprint: not peer-reviewed 10.1101/2021.02.23.432388. PMC9035045 34715055

[291] Khanna A., Côté A., Arora S., Moine L., Gehling V.S., Brenneman J., Cantone N., Stuckey J.I., Apte S., Ramakrishnan A. et al. Design, synthesis, and pharmacological evaluation of second generation EZH2 inhibitors with long residence time. ACS Med. Chem. Lett. 2020; 11:1205–1212. 32551002 10.1021/acsmedchemlett.0c00045 PMC7294713

[292] He Y., Selvaraju S., Curtin M.L., Jakob C.G., Zhu H., Comess K.M., Shaw B., The J., Lima-Fernandes E., Szewczyk M.M. et al. The EED protein–protein interaction inhibitor A-395 inactivates the PRC2 complex. Nat. Chem. Biol. 2017; 13:389–395. 28135237 10.1038/nchembio.2306

